# Activity-dependent glucose uptake in the mouse prefrontal cortex depends on endogenous beta-amyloid production

**DOI:** 10.1093/braincomms/fcag412

**Published:** 2026-10-28

**Authors:** Annamaria Fidilio, Sofia Varrasi, Rosa Santangelo, Filippo Caraci, Agata Copani

**Affiliations:** Department of Drug and Health Sciences, University of Catania, Catania 95125, Italy; Department of Drug and Health Sciences, University of Catania, Catania 95125, Italy; PhD Program in Neuroscience, University of Catania, Catania 95125, Italy; Department of Drug and Health Sciences, University of Catania, Catania 95125, Italy; Department of Drug and Health Sciences, University of Catania, Catania 95125, Italy; Oasi Research Institute-IRCCS, Unit of Neuropharmacology and Translational Neurosciences, Troina 94018, Italy; Department of Drug and Health Sciences, University of Catania, Catania 95125, Italy; Institute of Crystallography, National Research Council, Catania 95126, Italy

**Keywords:** beta-amyloid, prefrontal cortex, glucose uptake, activity-dependent metabolism, gamma-secretase

## Abstract

Beta-amyloid (Aβ) is continuously produced in the healthy brain as soluble monomers, yet its physiological function has remained largely unexplored compared to the well-studied toxic aggregates in Alzheimer's disease. We previously showed that depolarization-induced release of endogenous Aβ enhances glucose uptake in rodent cultured cortical neurons, an effect recapitulated by synthetic human Aβ_42_ monomers added to neurons upon glucose restoration after deprivation. To investigate whether this mechanism operates *in vivo*, we used memantine to transiently increase network activity in the prefrontal cortex of living mice. Memantine is known to disinhibit prefrontal cortex circuits by preferentially blocking NMDA receptors on GABAergic interneurons. In cultured cortical neurons, a brief challenge with memantine (10 μM) activated neuronal energy metabolism—glucose uptake and lactate release—through a γ-secretase-dependent mechanism. In the mouse prefrontal cortex, systemic memantine (20 mg/kg) increased 2-deoxyglucose uptake and concurrently enhanced γ-secretase activity and elevated soluble Aβ_42_ levels. Pre-treatment with the γ-secretase inhibitor DAPT (100 mg/kg) or with the selective Aβ_42_-lowering modulator JNJ-40418677 (80 mg/kg) prevented memantine-induced glucose uptake and reduced the memantine-induced increase in soluble Aβ_42_, demonstrating that acute Aβ_42_ production was required for activity-dependent glucose uptake in the mouse prefrontal cortex. This work establishes Aβ_42_ as a physiological neurometabolic coupler in the prefrontal cortex and has direct clinical implications for Alzheimer's disease: it supports therapeutic strategies aimed at preserving or restoring physiological Aβ_42_ levels rather than reducing them.

## Teaser

Fidilio *et al.* report that memantine-induced activation of the mouse prefrontal cortex increases local glucose uptake through acute beta-amyloid_42_ (Aβ_42_) production, as reducing Aβ_42_ levels with g-secretase inhibitors prevents this effect. This finding suggests that Aβ_42_ may act as a physiological neurometabolic coupler, a function potentially compromised in Alzheimer's disease.

## Graphical Abstract

**Graphical Abstract:**
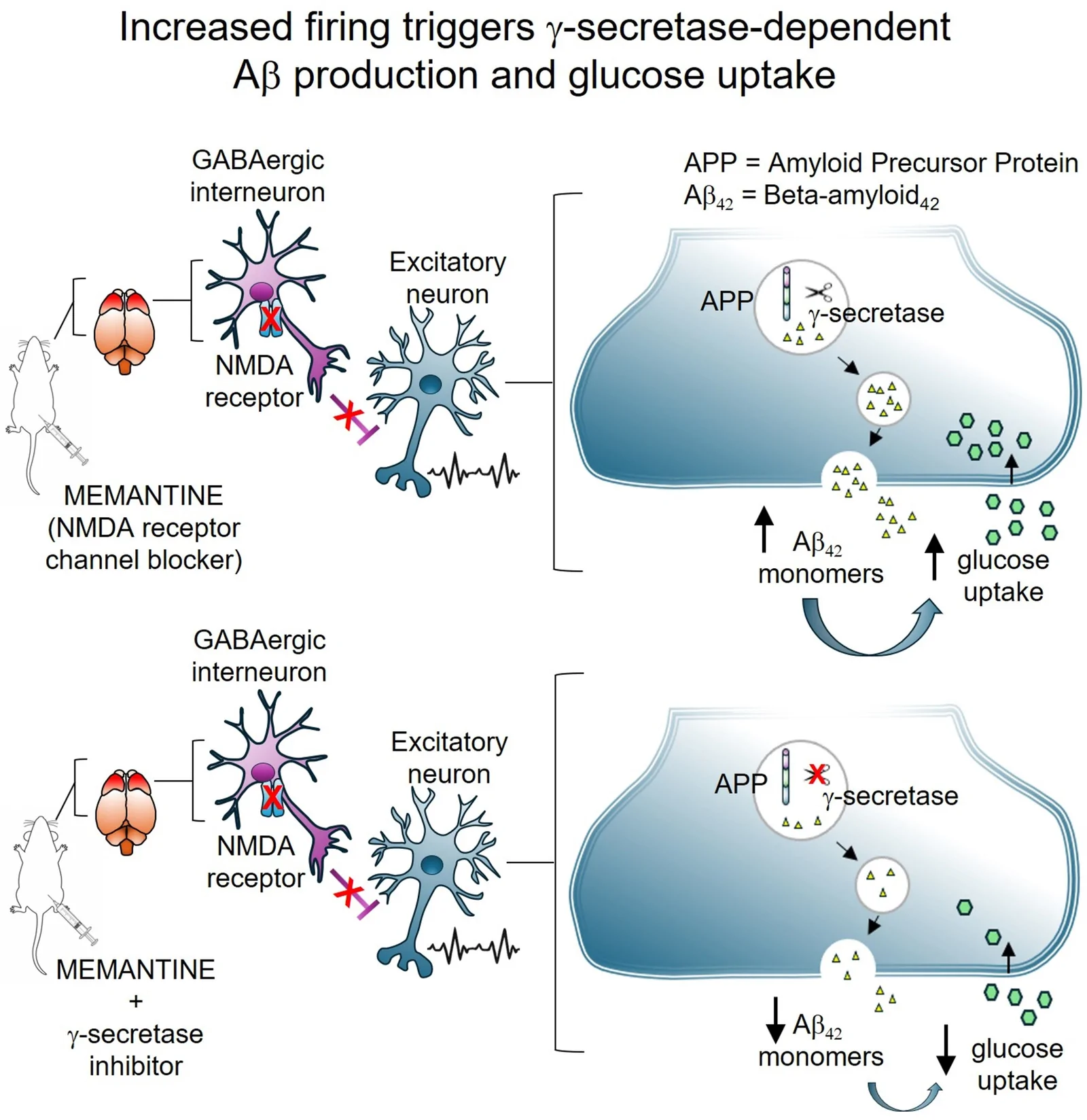

## Introduction

Aβ is continuously produced in the healthy brain through the sequential cleavage of the amyloid precursor protein by β- and γ-secretase.^1^ While the pathological aggregation of Aβ into oligomers and plaques has been extensively studied in AD, the physiological function of the monomeric form has remained largely unexplored.^2^ In pure cultures of rat or mouse cortical neurons, we have previously shown that a depolarization pulse with potassium or kainate enhances glucose uptake through the release of endogenous rodent Aβ, as this effect is prevented by γ-secretase inhibition and is absent in neurons from APP knockout mice, confirming that endogenous Aβ is required.^3,4^ Upon glucose restoration after deprivation in culture, the same increase in glucose uptake is recapitulated by synthetic human Aβ_42_ monomers, which activate type-1 insulin-like growth factor receptor (IGF-1R), drive GLUT3 translocation and promote aerobic glycolysis.^3-5^ Consistent with a loss-of-function mechanism—whereby pathological aggregation deprives neurons of the protective and metabolic actions of monomeric Aβ_42_—clinical trials in AD patients with secretase inhibitors, which reduce overall Aβ levels, have failed to show cognitive benefits, whereas anti-Aβ antibodies that selectively target aggregates while sparing monomers have shown efficacy.^6,7^

The PFC is a region of high metabolic demand and is particularly vulnerable to hypometabolism in AD.^8,9^ The PFC exhibits high baseline aerobic glycolysis, and the spatial distribution of this metabolic signature correlates with later Aβ deposition.^10^ However, whether activity-dependent Aβ release contributes to sustaining PFC energy metabolism remains unknown. To address this, we used memantine to transiently increase network activity in the living mouse PFC. Memantine disinhibits pyramidal neurons by preferentially blocking NMDA receptors on GABAergic interneurons.^11^ We therefore asked whether memantine-induced disinhibition triggers Aβ release and, in turn, enhances glucose uptake. Answering this question is relevant to understanding whether Aβ monomers support activity-dependent brain metabolism, a function that could be compromised in AD and other disorders characterized by PFC hypometabolism.

## Materials and methods

### Primary neuronal cultures: preparation and treatments

Animal care and experimentation were in accordance with national and institutional guidelines. Cultures of pure cortical neurons were obtained from rats at embryonic day 15 and grown as described previously.^5^ Cortical cells were plated onto 24-well plates pre-coated with 0.1 mg mL^−1^ poly-D-lysine (Sigma-Aldrich, Cat. No. P6407) and incubated at 37°C with 5% CO_2_ in a humidified atmosphere. Cytosine arabinoside (Ara-C, 5 μM) (Sigma Aldrich, Cat. No. C1768) was added to the cultures 18 h after plating to avoid the proliferation of non-neuronal elements and was kept for 3 days before partial medium replacement. Experiments were performed in mature culture at 14 days *in vitro* (DIV). All experiments were performed in artificial excitable CSF (aCSF) (3.5 mM KCl, 126 mM NaCl, 1.25 mM NaH2PO4, 0.5 mM MgSO4, 1 mM CaCl2, 26 mM NaHCO3) containing 10 μM glycine and, when required, 3 mM glucose. To block Aβ release, 100 nM γ-secretase inhibitor IX (DAPT) (Sigma-Aldrich, Cat No. 565770) was added to the artificial excitable CSF 2 h before the experiments. The experimental unit was the individual culture well. For *in vitro* experiments, culture wells were randomly assigned to treatment conditions. Researchers performing the glucose and lactate assays were blinded to treatment group allocation.

### *In vitro* glucose and lactate assays

Glucose content in the culture buffer (aCSF) was measured by Cayman’s Glucose Colorimetric Assay Kit (Cat. No. 10009582). Lactate release in the culture buffer was quantified by the Sigma-Aldrich Lactate Assay Kit (Cat. No. MAK 570, colorimetric detection). In both cases, following the technical instructions, absorbance was read by a spectrophotometric multiwell plate reader.

### Preparation of Aβ_42_ monomers

Aβ_42_ (HFIP-treated) was purchased from Bachem Distribution Services GmbH, Germany, dissolved at a 5 mM concentration in anhydrous dimethyl sulfoxide (DMSO) and stored at −20°C. At the time of its use, a solution of 100 μM Aβ in ice-cold DMEM F-12 was prepared and allowed to oligomerize overnight at 4°C. Monomers were isolated from the peptide suspension, containing both monomers and oligomers, by filtration through 10 kD cutoff filters as previously described.^5^

### Glucose uptake assessment in the mouse PFC

All experimental procedures were approved by the institutional animal care and use committee and authorized by the Italian Ministry of Health (authorization n. 149/2021-PR). Female 129/SVE mice (12 weeks old) were confirmed to be healthy and free of visible signs of disease prior to the experiments. The experimental unit was an individual mouse. Each determination represents a single animal, and determinations were collected across multiple independent experimental sessions. To avoid confounding cage activity with treatment effects, animals were allocated to treatment groups using a cage-blocked design: within each cage, littermates were randomly assigned to different experimental groups so that each cage contributed animals to multiple treatment conditions. This ensured that cage-level variability was balanced across groups. Researchers performing the 2-DG uptake measurements, western blot analysis and ELISA quantification were blinded to treatment group allocation. Blinding was maintained until all analyses were completed. No animals were excluded from the analysis. All animals that received the assigned treatments and completed the experimental protocol were included. Animals with visible signs of distress or injection site complications did not complete the experimental protocol. Sample sizes were determined based on previous *in vivo* studies from our laboratory and on practical constraints imposed by the time-critical nature of the 2-DG uptake procedure. No formal a priori power analysis was carried out.

Mice were fasted for 16 h prior to the experiment. When indicated, mice received an intraperitoneal (i.p.) injection of either the γ-secretase inhibitor IX, DAPT (100 mg/kg), or the γ-secretase modulator JNJ-40418677 (80 mg/kg) (MCE, Cat. No. HY-100604), or an equal volume of vehicle, at the doses previously reported to effectively modulate γ-secretase activity.^12,13^ Three hours later, animals were injected i.p. with memantine (20 mg/kg) (Sigma Aldrich, Cat. No. M9292) or saline; the memantine dose was selected based on previous studies demonstrating its central nervous system activity.^14^ Fifteen minutes after memantine administration, mice received an i.p. injection of 2-DG (3 mg/kg) (Sigma Aldrich, Cat. No. D8375) at a dose known to allow reliable detection of glucose uptake in brain tissue.^15^ Animals were euthanized 45 min after the 2-DG injection. The PFC was rapidly dissected; one hemisphere was immediately processed for 2-deoxyglucose-6-phosphate (2-DG6P) assessment, while the contralateral hemisphere was stored at −80°C for subsequent western blot analysis or ELISA quantification. For the 2-DG6P assessment, the dissected tissue was homogenized in 10 mM TRIS HCl (pH 8.1) and subsequently heated at 95°C for 15 min. Following centrifugation (18 000 × *g* for 15 min at 4°C) to remove the cell debris, the resulting supernatants were used to measure 2-DG uptake. The measurement of 2-DG uptake in the tissue was performed using the 2-DG uptake measurement kit (CSR-OKP-PMG-K01TE, Cosmo Bio, Tokyo, Japan) according to the manufacturer’s instructions, enabling the 2-DG uptake into the cells to be quantified as the amount of 2-DG6P without the interference of the remaining extracellular 2-DG based on the enzymatic photometric method.^16^

### Western blot analysis

PFC samples were homogenized in RIPA buffer supplemented with 1X protease (Sigma-Aldrich, Cat. No. P8340) and phosphatase inhibitors (Sigma-Aldrich, Cat. No. P5726 and P0044) using ULTRA-TURRAX homogenizer, then sonicated (three 10-second pulses on ice) and centrifuged at 14 000 × g for 15 min at 4°C. The supernatant was collected, and protein concentration was determined using Pierce^TM^ BCA Protein Assay Kit (ThermoFisher Scientific, Cat. No. 23227), measuring absorbance at 562 nm with SpectraMax® ABS Plus microplate reader (Molecular Devices, San Jose, CA, USA). Twenty mg of total protein per sample were separated on 10% or 4–12% Tris-glycine gels and transferred to PVDF membranes. Membranes were blocked in Intercept® (PBS) blocking buffer (LI-COR, Cat. No. 927-70003) for 1 h at room temperature, then incubated with anti-APP-C-terminal fragment (1:1000, BioLegend, Cat. No. 802801) and anti-actin (1:1000, Merck, Cat. No. A4700), as housekeeping, in blocking buffer at 4°C overnight. After washing in Tris-buffered saline (TBS) with 0.1% Tween 20, membranes were incubated with IRDye® 800CW goat anti-mouse IgG and 680LT goat anti-mouse IgG secondary antibodies (1:15000, LI-COR, Cat. No. 926-32210 and 926-68070, respectively) for 1 h at room temperature. Hybridization signals were detected using the Odyssey® Infrared Imaging System (LI-COR Biosciences, Lincoln, NE, USA).

### Extraction of soluble Aβ_42_ and ELISA quantification

Frozen PFC samples (30–40 mg) were homogenized using disposable pestles in 10 volumes (w/v) of ice-cold TBS supplemented with a protease inhibitor cocktail (Sigma-Aldrich P8340). The homogenate was centrifuged at 20 000 × g for 30 min at 4°C. The resulting supernatant (TBS-soluble fraction) was carefully collected, and protein concentration was determined using the Pierce^TM^ BCA Protein Assay Kit, measuring absorbance at 562 nm with SpectraMax® ABS Plus microplate reader. Aβ_42_ levels in the same fraction were quantified using a commercial ELISA kit specific for mouse Aβ_42_ (Invitrogen, Cat. No. KMB3441) according to the manufacturer’s instructions, and absorbance was measured at 450 nm using the same microplate reader. Aβ_42_ concentrations were normalized to total protein content as picograms of Aβ_42_ per microgram of sample protein.

### Statistical analysis

Statistical significance was assessed by one-way analysis of variance (ANOVA), followed by *post hoc* Fisher's LSD or Student-Newman-Keuls test for multiple comparisons, as appropriate. For comparisons between two groups, a two-tailed *t*-test was used. All analyses were performed using SigmaPlot 12.5.

## Results

### Memantine recapitulates the metabolic effects of excitation in cortical neurons

We previously demonstrated that a brief neuronal excitation with kainate (100 μM) stimulates glucose uptake and aerobic glycolysis through a mechanism requiring endogenous Aβ production.^4^ To test whether memantine, which increases neuronal activity by disinhibiting GABAergic interneurons,^14^ mimics this effect, we first examined its action on pure cortical neuronal cultures. Memantine (1, 4 and 10 μM) stimulated glucose uptake only at the highest concentration tested (data not shown). Specifically, following glucose deprivation and re-addition of 3 mM glucose, memantine (10 μM) significantly increased glucose consumption within 10 min (Fig. 1A) and lactate release within 40 min (Fig. 1B). Both effects were prevented by pre-incubation with the γ-secretase inhibitor IX, DAPT (100 nM), indicating that memantine promotes glucose uptake and aerobic glycolysis through a γ-secretase-dependent pathway, mirroring the effect of kainate-induced excitation.

**Figure 1:**
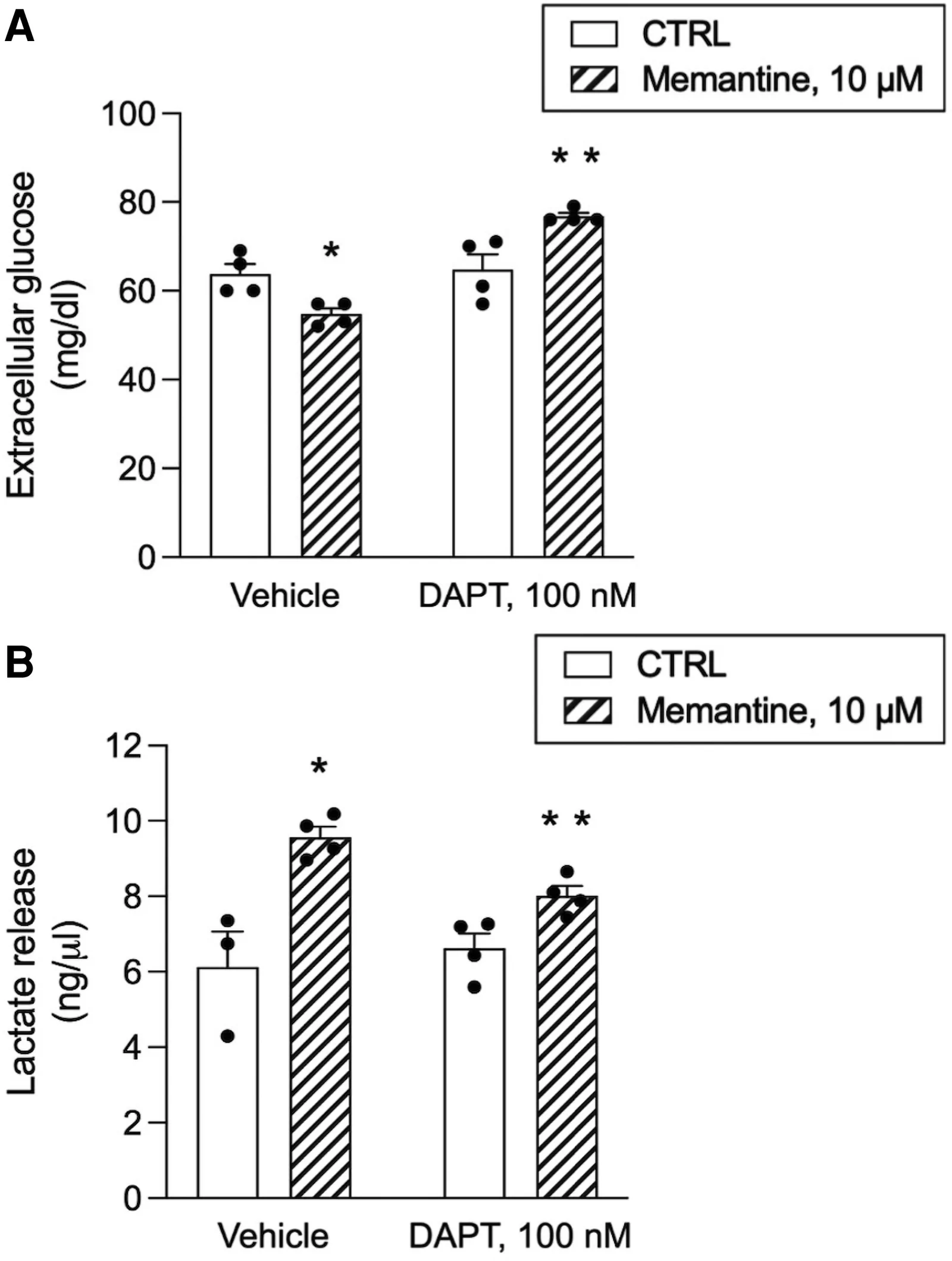
**Memantine promotes glucose uptake and lactate release in cultured rat cortical neurons via a γ-secretase-dependent pathway**. Following 2 h of glucose deprivation, 3 mM glucose was added to neuronal cultures. A treatment with memantine (10 μM) stimulated glucose uptake after 10 min (**A**) and lactate release after 40 min (**B**). Glucose consumption was measured as glucose (mg/dl) remaining in the incubation buffer 10 min following re-addition. With respect to the initial 3 mM glucose concentration, no glucose uptake occurred within 10 min unless memantine was added. The γ-secretase inhibitor IX (DAPT, 100 nM) prevented memantine-stimulated glucose uptake (**A**) and lactate release at 40 min (**B**). Bars represent the means ± SEM of three to four determinations. Each data point represents an independent measure from a representative experiment. In A, *P* < 0.001 versus *control (CTRL) or **memantine alone. In B, *P* < 0.001 versus *CTRL or *P* < 0.05 versus **memantine alone; one-way ANOVA with *post hoc* Fisher LSD multiple comparison method.

### Memantine increases glucose uptake and activates γ-secretase in the PFC *in vivo*

We next examined whether memantine enhances glucose uptake in the living brain. A single intraperitoneal injection of memantine (20 mg/kg) in 12-week-old female mice significantly increased 2-DG6P accumulation in the PFC compared to saline-injected controls (Fig. 2A). To determine whether memantine activates γ-secretase in the PFC, we measured APP C-terminal fragment (APP-CTF) levels. Because APP-CTFs are direct substrates of γ-secretase, their accumulation indicates reduced γ-secretase activity, whereas decreased levels reflect enhanced γ-secretase cleavage.^17^ Memantine treatment reduced APP-CTF levels compared to controls (Fig. 2B), consistent with enhanced γ-secretase cleavage of APP.

**Figure 2:**
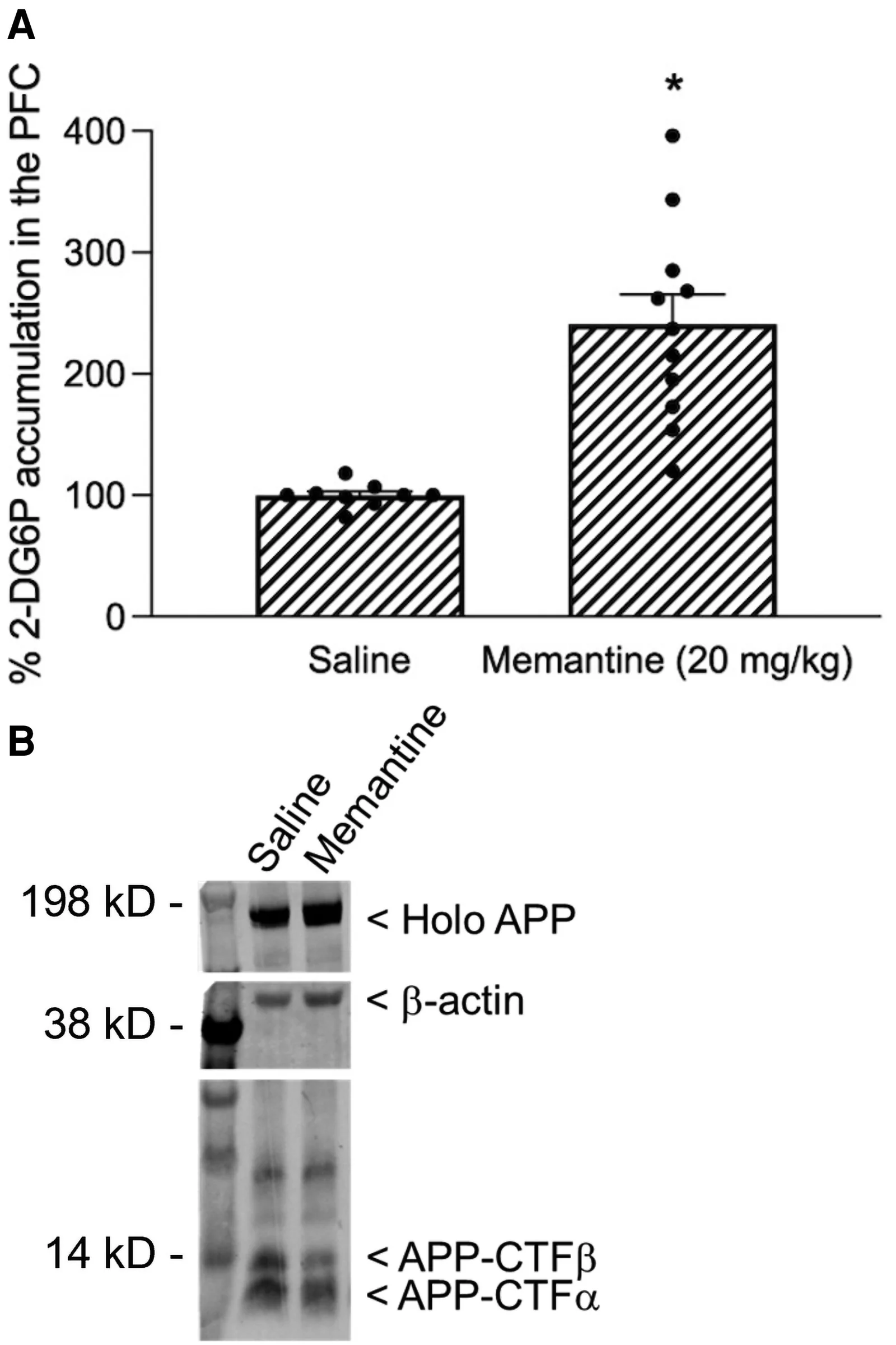
**Memantine enhances glucose uptake and stimulates γ-secretase activity in the mouse PFC**. (**A**) Increased PFC glucose uptake after memantine. A single injection of memantine led to significantly higher 2-DG6P levels in 12-week-old female SVE mice within 60 min. Bars represent means ± SEM of nine to 11 determinations from five experiments. Each data point represents an independent measure from five experiments. Within each experiment, the mean value of the saline condition was set to 100%, and each individual data point was normalized accordingly; these normalized values were then pooled across experiments, so that data are expressed as percentage of 2-DG6P accumulation relative to saline. Basal levels of 2-DG6P in the PFC under control conditions (saline-injected mice) ranged from 0.05 to 3.7 pmol/μg protein across experiments, reflecting the inherent variability of *in vivo* measurements. *P < 0.05 versus saline by two-tailed *t*-test. **(B**) Western blot analysis of PFC lysates from memantine-treated mice showed reduced levels of APP C-terminal fragments (APP-CTF), indicating enhanced γ-secretase activity. Full western blot images can be found in Supplementary material. Holo APP, full length APP. The 42 kD band of β-actin is shown *as a* loading control. The slight increase in protein loading in the memantine-treated sample explains the corresponding increase in holo-APP levels. Nevertheless, APP-CTF levels were reduced after memantine treatment. PFC, prefrontal cortex. 2-DG6P, 2-deoxyglucose-6-phosphate.

### Blockade of γ-secretase prevents memantine-induced cerebral glucose uptake

If memantine-induced glucose uptake depends on γ-secretase activity, pharmacological inhibition of the enzyme should abolish the effect. Pre-treatment with the γ-secretase inhibitor IX, DAPT (100 mg/kg), three hours before the memantine challenge significantly reduced 2-DG6P accumulation in the PFC (Fig. 3A). DAPT-treated mice showed increased APP-CTF levels compared to mice receiving memantine alone (Fig. 3B), confirming effective γ-secretase blockade. These results demonstrate that memantine-induced cerebral glucose uptake requires active γ-secretase and, consequently, activity-dependent Aβ production.

**Figure 3:**
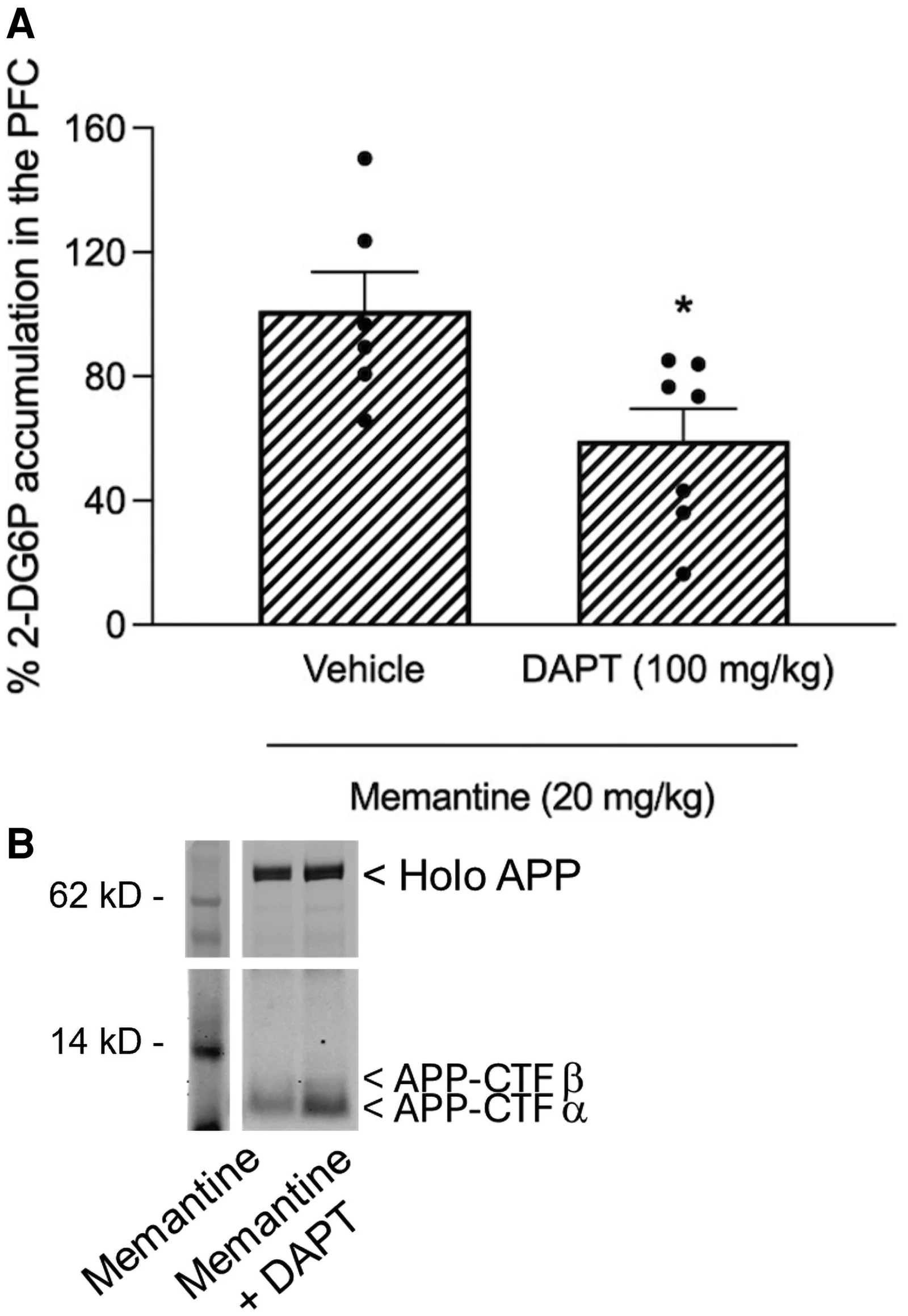
**Inhibition of γ-secretase by DAPT reduces memantine-induced glucose uptake in the mouse PFC**. (**A**) Pre-treatment with DAPT reduced glucose uptake in the mouse PFC following a memantine challenge. Bars represent means ± SEM of six to seven determinations from three experiments. Each data point represents an independent measure from three experiments. Within each experiment, the mean value of the memantine-treated condition was set to 100%, and each individual data point was normalized accordingly. These normalized values from all experiments were then pooled, so that data are expressed as percentage of 2-DG6P accumulation relative to memantine. *P < 0.05 versus memantine alone by two-tailed *t*-test. (**B**) Western blot analysis of PFC lysates from DAPT-pretreated, memantine-injected mice showed increased levels of APP C-terminal fragments (APP-CTF), indicating reduced γ-secretase activity. Full western blot images can be found in Supplementary material. Holo APP, full length APP. PFC, prefrontal cortex. 2-DG6P, 2-deoxyglucose-6-phosphate.

### Selective lowering of Aβ_42_ by a γ-secretase modulator blunts the metabolic response to memantine

To determine whether the effect of memantine specifically requires the Aβ_42_ isoform, we used the γ-secretase modulator JNJ-40418677, which selectively reduces Aβ_42_ without affecting Notch processing.^12^ Mice pre-treated with JNJ-40418677 (80 mg/kg) three hours before memantine showed a marked reduction in memantine-stimulated 2-DG6P accumulation in the PFC (Fig. 4A). Under the same experimental conditions, JNJ-40418677 pre-treatment also significantly reduced soluble Aβ_42_ levels in the PFC compared to memantine alone, as measured by ELISA (Fig. 4B). The absolute level in control mice was 0.011 pg/μg protein, and the normalized data show that memantine increased soluble Aβ42 relative to control, an effect prevented by JNJ-40418677 (Fig. 4B). These findings indicate that memantine-induced PFC glucose uptake depends on the availability of soluble Aβ_42_, supporting a role for the monomeric peptide in activity-dependent metabolic coupling.

**Figure 4:**
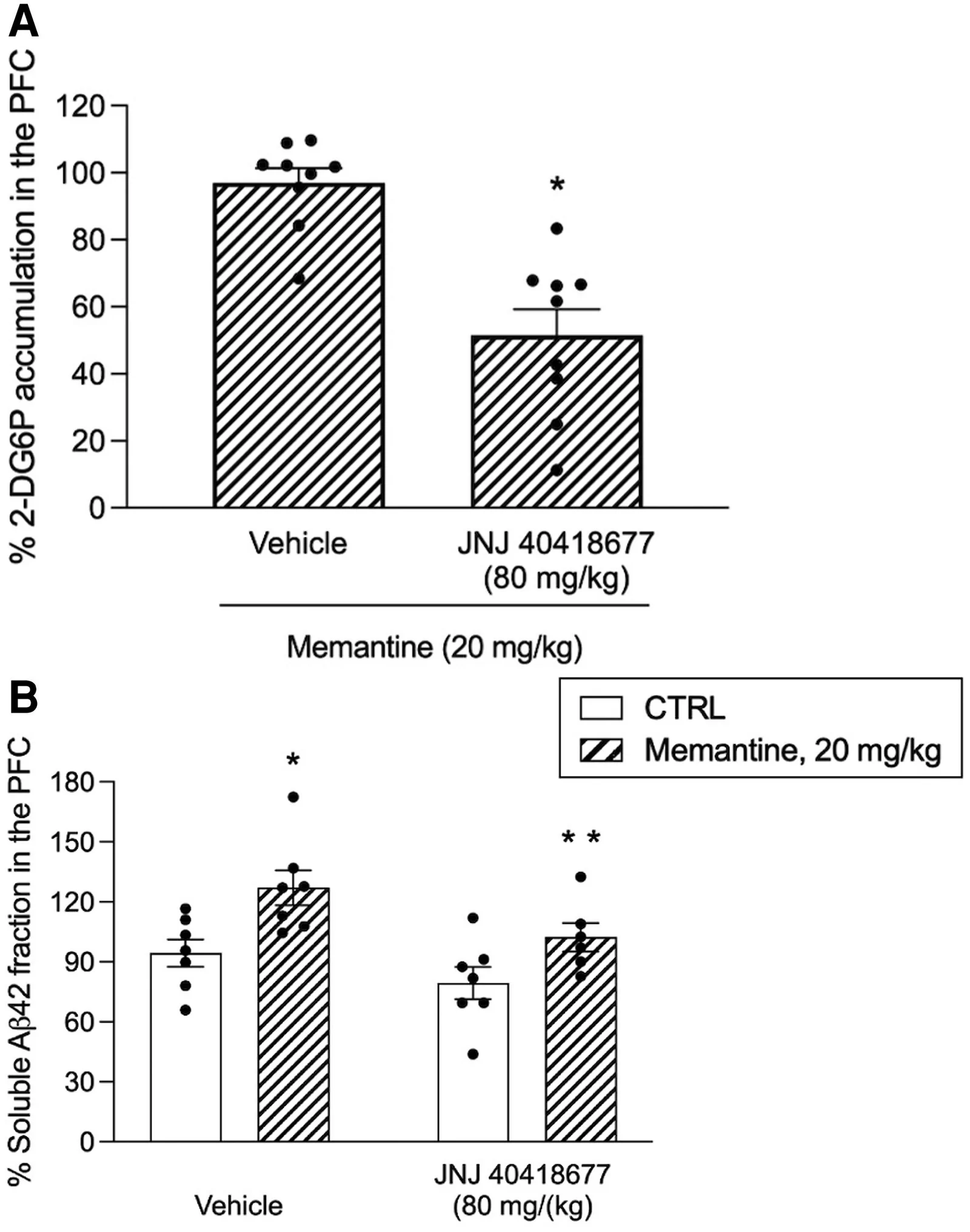
**The selective Aβ42-lowering γ-secretase modulator, JNJ 40418677, reduces memantine-induced cerebral glucose uptake in the mouse PFC**. (**A**) Pre-treatment with JNJ 40418677 reduced glucose uptake in the mouse PFC following a memantine challenge. Bars represent means ± SEM of nine determinations from three experiments. Each data point represents an independent measure from three experiments. Within each experiment, the mean value of the memantine-treated condition was set to 100%, and each individual data point was normalized accordingly. These normalized values from all experiments were then pooled, so that data are expressed as percentage of 2-DG6P accumulation relative to memantine. *P < 0.001 versus memantine alone by two-tailed *t*-test. (**B**) Soluble Aβ_42_ levels were reduced in the PFC of JNJ 40418677-pretreated, memantine-injected mice, as assessed by ELISA. The mean level of soluble Aβ_42_ in control (saline-injected) mice was 0.011 pg/μg protein. Bars represent means ± SEM of six to seven determinations from two experiments. Within each experiment, the mean value of the control condition was set to 100%, and each individual data point was normalized accordingly; these normalized values were then pooled across experiments, so that data are expressed as percentage of soluble Aβ_42_ relative to control. *P* < 0.05 versus *control (CTRL) or **memantine alone; one-way ANOVA with *post hoc* Student-Newman-Keuls for multiple comparison method. PFC, prefrontal cortex. 2-DG6P, 2-deoxyglucose-6-phosphate.

## Discussion

The PFC is a region of high metabolic demand and is particularly vulnerable to hypometabolism in AD.^8,9^ The PFC exhibits high baseline aerobic glycolysis, and the spatial distribution of this metabolic signature correlates with later Aβ deposition.^10^ We therefore set out to investigate the mechanisms that support activity-dependent energy metabolism in this region. Female mice were used in this study as a first step toward future experiments in the CRND8 transgenic mouse model of AD.^18^ In this model, females develop an aggressive pathology^19^ and, in the future, will be used to investigate neurometabolic coupling across the progression of AD pathology, from the pre-plaque stage to plaque deposition within 16 weeks.^18^

Importantly, the present finding that identifies Aβ_42_ as a physiological neurometabolic coupler in the PFC stands on its own as a discovery, independent of any future translational and clinical applications.

To probe activity-dependent metabolic coupling in the PFC, we needed a pharmacological tool that could reliably and transiently increase network activity. Memantine, an uncompetitive NMDA receptor antagonist currently used for the treatment of AD, is known to disinhibit pyramidal neurons by preferentially blocking NMDA receptors on GABAergic interneurons.^14^ We reasoned that memantine-induced disinhibition would increase presynaptic activity and, consequently, trigger activity-dependent Aβ release. We first tested *in vitro* whether memantine could stimulate glucose uptake and metabolism (i.e. aerobic glycolysis) in an Aβ-dependent manner, using pure cortical neuronal cultures. We tested memantine at 1, 4 and 10 μM; only the highest concentration was found to be effective. This concentration is commonly used *in vitro* to achieve NMDA receptor blockade^20^ while preserving fast off-rate kinetics.^21^ Using this concentration, memantine stimulated glucose uptake and lactate release in a γ-secretase-dependent manner, recapitulating our previous findings with kainate-induced depolarization in cultured neurons.^4^

We then translated these observations to the living brain, showing that memantine administered systemically at a dose effective for brain activation (20 mg/kg)^14^ increased 2-DG uptake in the mouse PFC and reduced APP C-terminal fragment levels, consistent with enhanced γ-secretase activity.^17^ While APP-CTF levels are a reasonable surrogate marker of γ-secretase activity, we acknowledge that their abundance may also be influenced by additional mechanisms affecting APP processing or turnover. Nevertheless, the consistency of the APP-CTF changes with the functional outcomes supports our interpretation. The finding that the γ-secretase inhibitor DAPT prevented memantine-induced glucose uptake both *in vitro* and *in vivo* directly links the metabolic response to ongoing Aβ production. To determine whether the effect specifically required the Aβ_42_ isoform, we used the γ-secretase modulator JNJ-40418677, which selectively lowers Aβ_42_ without affecting Notch processing.^12^ Pre-treatment with JNJ-40418677 blunted memantine-induced glucose uptake in the PFC and concomitantly reduced soluble Aβ_42_ levels, as measured by ELISA. The mean level of soluble Aβ_42_ in the control PFC was 0.011 pg/μg protein, which falls within the low picogram range expected for endogenous Aβ in wild-type mouse brain.^22^ The observation that JNJ-40418677, which reduces Aβ_42_ without altering total Aβ or Notch signalling, phenocopied the effect of DAPT strongly implicates the monomeric Aβ_42_ as the critical effector of memantine-induced metabolic activation. We acknowledge a limitation of our *in vitro* experiments: we did not directly measure Aβ release following memantine challenge in cultured neurons. However, we addressed this directly *in vivo* by quantifying soluble Aβ42 levels in the PFC. As shown in Fig. 4B, memantine challenge increased soluble Aβ42 levels in the PFC of control mice, and this increase was significantly reduced by pre-treatment with the selective Aβ42-lowering modulator JNJ-40418677. Under the same experimental conditions, JNJ-40418677 also significantly reduced memantine-induced glucose uptake (Fig. 4A). Thus, the same pharmacological manipulation that lowers Aβ42 production also blunts the metabolic response, providing a direct link between Aβ42 production and glucose uptake in the PFC. The convergent evidence from two pharmacologically distinct agents, DAPT and JNJ-40418677, strongly supports the conclusion that acute Aβ42 production is required for memantine-induced glucose uptake. We also recognize that our mechanistic conclusions rely exclusively on pharmacological approaches. While the use of DAPT and JNJ-40418677 is appropriate, pharmacological manipulation alone cannot unequivocally establish causality. Genetic approaches, such as conditional APP knockout, would provide complementary evidence in future studies.

For the *in vivo* experiments, we used a non-radioactive enzymatic method for 2-DG6P quantification that is sensitive and reliable^16^ but requires rapid tissue processing. The time-critical nature of the procedure necessarily limits the number of animals that can be processed per experimental session. Consequently, determinations were collected across multiple independent sessions, and the consistency of the observed effects supports the robustness of the findings despite constraints on group size. We also acknowledge that the 2-DG method measures total tissue glucose uptake, encompassing both neuronal and glial contributions. However, the prevention of memantine-induced glucose uptake by γ-secretase inhibitors points to acute, activity-dependent Aβ production. Although glial cells can produce Aβ—primarily as N-terminally truncated species^23—^and are sensitive to γ-secretase inhibitors,^24,25^ their Aβ production typically occurs in response to pathological or inflammatory stimuli and unfolds over hours rather than minutes.^25^ Therefore, the rapid (minutes) time course of the memantine challenge favours activity-dependent neuronal release. This interpretation is reinforced by our experiments in pure neuronal cultures, in which the same γ-secretase-dependent response to memantine occurs without any glial contribution. While we cannot formally exclude a minor astrocytic contribution to the total glucose uptake measured *in vivo*, the rapid blockade by γ-secretase inhibition and the recapitulation of the effect in pure neuronal cultures indicate that the critical signal is neuronal. The demonstration that memantine-induced PFC glucose uptake requires soluble Aβ_42_ raises the question of whether distinct pathological conditions—including AD,^8,9^ late-life depression,^26^ frontotemporal dementia,^27^ and normal ageing^28^—converge on failure of this Aβ-dependent metabolic coupling. If so, the consequence could be a common inability to adequately fuel PFC neurons during periods of high demand, suggesting that impairment of this pathway might represent a shared endpoint across multiple disorders. The current paradigm for activity-dependent metabolic support is the astrocyte-neuron lactate shuttle (ANLS), in which astrocytic glycolysis produces lactate that is shuttled to neurons to fuel oxidative phosphorylation.^29^ The mechanism we describe appears to be largely neuron-autonomous and is triggered by presynaptic Aβ release following increased network activity. Rather than replacing the ANLS, this intrinsic system may complement it by providing rapid local energy support during sustained firing, potentially before glial responses are fully engaged. However, we acknowledge that the 2-DG method measures total tissue glucose uptake, and we cannot exclude the possibility of additional glial contributions, particularly at later time points.

The clinical implications of these findings are twofold. First, they are consistent with a loss-of-function hypothesis for AD, wherein the loss of functional, soluble Aβ_42_—whether through aggregation into amyloid or through degradation by endopeptidases such as neprilysin—could contribute to neuronal dysfunction independently of, or in addition to, the accumulation of insoluble amyloid.^30^ Our data demonstrate that acute Aβ_42_ production is required for activity-dependent PFC glucose uptake, raising the possibility that depletion of the functional monomer pool may impair neuronal energy metabolism. Second, these findings caution against therapeutic strategies aimed at lowering Aβ_42_ levels and suggest that the paradigm of simply reducing Aβ burden in AD may warrant reconsideration.

## Supplementary Material

fcag412_Supplementary_Data

## Data Availability

Data are available from the corresponding author upon request.
