# Supplementary material for: Activity-dependent glucose uptake in the mouse prefrontal cortex depends on endogenous beta-amyloid production
Source: Brain Commun. 2026 Oct 28;8(6):fcag412. doi: 10.1093/braincomms/fcag412 (PMC13739058; doi:10.1093/braincomms/fcag412)
Supplement: Supplementary file 1 [file fcag412_supplementary_data.zip › Original_submission_decision_letter.pdf]

From: brcoms.editorialoffice@paeditorial.co.uk

To:

CC:

BCC:

Subject: Brain Communications - Decision on Manuscript ID BRAINCOM-2026-768

Body: 16-Jul-2026

Dear Prof. COPANI,

Thank you for your recent submission BRAINCOM-2026-768 entitled "Activity-dependent glucose uptake in the mouse prefrontal cortex depends on endogenous Aβ production" to Brain Communications.

Your manuscript has been reviewed, and while the reviewers and editorial team appreciate many aspects of the paper, they raise some concerns that you will need to address in order for the paper to be considered for publication.

If you can address these concerns, I invite you to submit a revised manuscript and a response to each of the reviewers' comments. The reviewer(s)' comments are included at the bottom of this message. Please pay special attention to the first comment of Reviewer 2 as it represents a mechanistic weakness that must be addressed and/or discussed.

In addition to the reviewers' comments, please also address any comments from our Scientific Editor added below to comply with our requirements for presentation of scientific data at Brain Communications. Please note we will not accept the paper unless you comply with our policies on data analysis and presentation as noted in the comments from the Scientific Editor.

Please format your revised manuscript according to our Instructions to Authors
https://academic.oup.com/braincomms/pages/General_Instructions
A summary of key points is attached to help with this. Please note that there can be substantial delays to publication if you don't resubmit in line with our requirements.

REMINDER: if you have not already done so, when submitting your revised manuscript, please include a short alternative-text description under the figure legend for each figure. Alt-text is required to ensure the accessibility of our publications. Guidance is available on the journal's Author Guidelines webpage and at https://academic.oup.com/pages/for-authors/journals/preparing-and-submitting-your-manuscript

To revise your manuscript, click the link below or log into https://mc.manuscriptcentral.com/braincom and enter your Author Centre, where you will find your manuscript title listed under "Manuscripts with Decisions." Under "Actions," click on "Create a Revision." Your manuscript number will be appended to denote a revision.

*** PLEASE NOTE: This is a two-step process. After clicking on the link, you will be directed to a webpage to confirm. ***

https://mc.manuscriptcentral.com/braincom?URL_MASK=c478e305535a481bba24ea8a93d39fd9

Please upload a revised manuscript with changes tracked with track changes or in coloured text.

When submitting your revised manuscript, you will be asked to respond to the reviewer(s) comments. Please be as specific as possible in your response to all of the comments.

The editorial team at Brain Communications aims to increase transparency in scientific publishing and to demonstrate the value that peer review brings to the scientific process. Please therefore consider whether you are willing to have reviewer(s)' comments on your paper and your response published as supplementary data with the paper if it is accepted. This is entirely optional and will only be done if both the authors and reviewers agree. While submitting your revision there will be an opportunity to review or update the answer you gave previously, if necessary.

IMPORTANT: Your original files are available to you when you upload your revised manuscript. Please delete any redundant files before completing the submission.

Because we are trying to facilitate timely publication of manuscripts submitted to Brain Communications, we would appreciate your revised manuscript as soon as possible. If it is not possible for you to resubmit within 3 months, the option to submit a revision will expire, and we would then consider your revised paper as a new submission. However, if you need a little more time, please get in touch and we can extend this deadline for you.

Please note that Brain Communications is a fully open access journal, and if your article is accepted for publication, an open access charge will be payable (this does not apply to letters and scientific commentaries). Details can be found in our Instructions for Authors: https://academic.oup.com/braincomms/pages/General_Instructions

Once again, thank you for submitting your manuscript to Brain Communications and I look forward to receiving your revision.

Best wishes

Dr. Alejandro Higuera-Matas
Associate Editor, Brain Communications

Prof. David Belin
Editor-in-Chief Brain Communications

Reviewer(s)' Comments to Author:

Reviewer: 1

Comments to the Author
The authors asked whether activity-dependent Aβ release contributes to sustaining PFC energy metabolism using cultures of pure cortical neurons obtained from rats at embryonic day 15 exposed to memantine to transiently increase network activity. Memantine triggered Aβ release and increased glucose uptake through a γ-secretase-dependent pathway (the effect was abolished using a γ-secretase inhibitor). Importantly, the authors determined that the effect of memantine specifically requires the Aβ42 isoform as this effect was reduced by using the γ-secretase modulator JNJ-40418677, which selectively lowers Aβ42 without affecting Notch processing.

The study is well executed and the findings relevant. I suggest the authors clarify in the abstract and discussion the major clinical implication: these findings argue for moving gamma-secretase modulation efforts from lowering to increasing gamma-secretase activity and Aβ42 levels. This is more important than the current conclusion, namely that the physiological function of Aβ42 "may be compromised in AD and other disorders characterized by PFC hypometabolism." This, in fact, may be included in the discussion but is not the most critical implication.

The results provide an explanation not only for the worsening experienced by patients exposed to gamma-secretase inhibitors (several trials over the years) and to gamma-secretase modulators tarenflurbil, all of which were designed to lower Aβ42 levels. The Notch-sparing Aβ42-reducing gamma-secretase modulators RG6289 and RO7269162 are currently under evaluation, with results expected next year. It is possible that future molecules with similar intent will continue to be tested despite negative findings, hence the importance of including the implications.

Consider adding the specific levels of Aβ42 in the appropriate figures (e.g. Fig 4) and in the corresponding text.

Lastly, consider noting in the discussion that these results support a loss of function hypothesis for Alzheimer's disease where the loss of functional, soluble Aβ42 (both through an endopeptidase, neprilysin, and as it aggregates into amyloid) may be more detrimental to the brain than the corresponding accrual of insoluble amyloid (PMID: 38270185).

Reviewer: 2

Comments to the Author
This is manuscript proposes a physiological role for endogenous Aβ42 in activity-dependent glucose uptake in the healthy prefrontal cortex. The study is conceptually novel and challenges the traditional view of Aβ solely as a pathogenic peptide, making it of broad interest to the neuroscience and Alzheimer's disease communities.

My main concern is that the experimental evidence does not yet fully support the strength of the

central mechanistic conclusions.

First, the manuscript does not directly demonstrate that memantine increases endogenous Aβ42 production or release. This is a key assumption of the proposed model, yet the conclusion is inferred from γ-secretase inhibition and modulation rather than from direct measurement of Aβ42 following memantine treatment. Demonstrating such an increase, or alternatively moderating the conclusions, would substantially strengthen the manuscript.

Second, APP-CTF levels are interpreted as evidence of altered γ-secretase activity. Although this is a reasonable surrogate marker, APP-CTF abundance may also be influenced by additional mechanisms affecting APP processing or turnover. This limitation should be acknowledged.

Third, the mechanistic conclusions rely exclusively on pharmacological approaches. While the use of DAPT and JNJ-40418677 is appropriate, pharmacological manipulation alone cannot unequivocally establish causality. This limitation should also be discussed.

Finally, glucose uptake is quantified in whole prefrontal cortex tissue. Since the assay cannot distinguish neuronal from glial glucose utilization, I believe statements suggesting a neuron-autonomous mechanism are stronger than warranted. A more balanced discussion acknowledging possible contributions from astrocytes and other brain cells would improve the manuscript.

Comments from Scientific Editor Team

-Please add an Abbreviated Summary (up to 50 words) of your paper that captures the main purpose and conclusions of the work. The summary should state the main results written in the third person perspective ('[1st author surname] et al. report that ...') and conclusion. This summary will be used in the contents list online and should not contain any abbreviations other than those on the accepted abbreviations list and accepted gene/protein names, making it accessible to the non-specialist. Please upload it as a Word document. Allowed abbreviations are: AIDS; ANOVA; AMPA; ATP; A,T,C,G; CNS; CSF; CT; DNA; ECG; EEG; EMG; GABA; HIV; MRI; NMDA; PET; PCR; RNA.

-Please provide a Graphical Abstract. A graphical abstract is a visual representation of the central finding or methodology of the paper. It will be displayed in the table of contents. Authors should strive to make the graphical abstract informative, interesting, visually appealing, and straightforward (no caption needed, no abbreviations except gene names). The graphical abstract must not contain any elements for which you do not hold the copyright, such as figures or images from other articles or websites etc. The technical requirements for the graphical abstracts are:
- Size: 120 x 120 mm (300-600dpi) or 3000 x 3000 pixels
- Font: Arial 12-16 pt
- File type: tiff or PDF
- Aspect ratio: 1:1 preferred

- Please note our policy on code availability: If any codes were generated for data analysis for this manuscript (e.g. using R, MATLAB, or similar), please make this code available either via an

online repository (and provide a link within the data availability statement), or within the manuscript.

-As a reminder at this revision stage, if you plan to edit the current figures and tables, or to make new figures and tables, please ensure that these items fit a typeset journal page (the area available for a figure or table is 170 mm x 210 mm), or are uploaded as supplementary material. Please view your figures at this size and make sure that all lettering and text are large enough to read.

- Please include the appropriate reporting guideline checklist to comply with our ethical standards (see Reporting Guideline section within 'Instructions to authors'), or since this is a registered report at stage 1, please report the name of the reporting guideline checklist that the authors plan to supply to the stage 2.

- For each statistical analysis as part of a figure or table, please make sure to report in the legend, where missing, the test used and relevant test statistic values alongside p values (for example F or t values for ANOVAs and t-tests). Also, please, provide details of the experimental unit for each group analysed in these plots, and if the number is <10 show the single data-points.   Where data-points are shown, please state what each data-point represents.
