# Supplementary material for: Activity-dependent glucose uptake in the mouse prefrontal cortex depends on endogenous beta-amyloid production
Source: Brain Commun. 2026 Oct 28;8(6):fcag412. doi: 10.1093/braincomms/fcag412 (PMC13739058; doi:10.1093/braincomms/fcag412)
Supplement: Supplementary file 1 [file fcag412_supplementary_data.zip › Original_submission_manuscript.pdf]

Brain Communications

Activity-dependent glucose uptake in the mouse prefrontal cortex depends on endogenous Aβ production

Journal:	*Brain Communications*
Manuscript ID	BRAINCOM-2026-768
Manuscript Type:	Report
Date Submitted by the Author:	17-Jun-2026
Complete List of Authors:	Fidilio, Annamaria; University of Catania, Drugs and Health Sciences<br>Varrasi, Sofia; University of Catania, Drugs and Health Sciences<br>Caraci, Filippo; University of Catania, Drugs and Health Sciences<br>COPANI, Agata; University of Catania, Drugs and Health Sciences
Keywords:	beta-amyloid, prefrontal cortex, glucose uptake, activity-dependent metabolism, gamma secretase

SCHOLARONE
MANUSCRIPTS

Activity-dependent glucose uptake in the mouse prefrontal cortex depends on endogenous Aβ production

Annamaria Fidilio[1], Sofia Varrasi[1,2], Filippo Caraci[1,3], Agata Copani[1,4]

[1]Department of Drug and Health Sciences, University of Catania, 95125 Catania, Italy.

[2]PhD Program in Neuroscience, University of Catania.

[3]Oasi Research Institute-IRCCS, Unit of Neuropharmacology and Translational Neurosciences, 94018 Troina, Italy.

[4]Institute of Crystallography, National Research Council, 95126 Catania, Italy.

Correspondence to: Agata Copani, Department of Drug and Health Sciences, University of Catania, 95125 Catania, Italy. Tel: + 39(095)7384212. E-mail: acopani@katamail.com.

For Review Only

Abstract

Beta-amyloid (Aβ) is continuously produced in the healthy brain as soluble monomers, yet its physiological function has remained largely unexplored compared to the well-studied toxic aggregates in Alzheimer's disease (AD). We previously showed that depolarization-induced release of endogenous Aβ enhances glucose uptake in rodent cultured cortical neurons, an effect recapitulated by synthetic human $A\beta_{42}$ monomers added to neurons upon glucose restoration after deprivation. To investigate whether this mechanism operates in vivo, we used memantine to transiently increase network activity in the prefrontal cortex (PFC) of living mice. Memantine is known to disinhibit PFC circuits by preferentially blocking NMDA receptors on GABAergic interneurons. In cultured cortical neurons, a brief challenge with memantine (10 μM) activated neuronal energy metabolism - glucose uptake and lactate release - through a γ-secretase-dependent mechanism. In the mouse PFC, systemic memantine (20 mg/kg) increased 2-deoxyglucose (2-DG) uptake and concurrently enhanced γ-secretase activity. Pre-treatment with the γ-secretase inhibitor DAPT (100 mg/kg) or with the selective $A\beta_{42}$-lowering modulator JNJ-40418677 (80 mg/kg) prevented memantine-induced glucose uptake, demonstrating that acute $A\beta_{42}$ production was required for activity-dependent glucose uptake in the mouse PFC. This work establishes $A\beta_{42}$ as a physiological neurometabolic coupler in the PFC, a function that may be compromised in AD and other disorders characterized by PFC hypometabolism.

Keywords: beta-amyloid, prefrontal cortex, glucose uptake, activity-dependent metabolism, gamma-secretase

For Review Only

Introduction

Aβ is continuously produced in the healthy brain through the sequential cleavage of the amyloid precursor protein by β- and γ-secretase[1]. While the pathological aggregation of Aβ into oligomers and plaques has been extensively studied in AD, the physiological function of the monomeric form has remained largely unexplored[2]. In pure cultures of rat or mouse cortical neurons, we have previously shown that a depolarization pulse with potassium or kainate enhances glucose uptake through the release of endogenous rodent Aβ, as this effect is prevented by γ-secretase inhibition and is absent in neurons from APP knockout mice, confirming that endogenous Aβ is required[3-4]. Upon glucose restoration after deprivation in culture, the same increase in glucose uptake is recapitulated by synthetic human $A\beta_{42}$ monomers, which activate type-1 insulin-like growth factor receptor (IGF-1R), drive GLUT3 translocation, and promote aerobic glycolysis[5, 3-4]. Consistent with a loss-of-function mechanism - whereby pathological aggregation deprives neurons of the protective and metabolic actions of monomeric $A\beta_{42}$ - clinical trials in AD patients with secretase inhibitors, which reduce overall Aβ levels, have failed to show cognitive benefits, whereas anti-Aβ antibodies that selectively target aggregates while sparing monomers have shown efficacy[6-7].

The PFC is a region of high metabolic demand and is particularly vulnerable to hypometabolism in AD[8-9]. The PFC exhibits high baseline aerobic glycolysis, and the spatial distribution of this metabolic signature correlates with later Aβ deposition[10]. However, whether activity-dependent Aβ release contributes to sustaining PFC energy metabolism remains unknown. To address this, we used memantine to transiently increase network activity in the living mouse PFC. Memantine disinhibits pyramidal neurons by preferentially blocking NMDA receptors on GABAergic interneurons[11]. We therefore asked whether memantine-induced disinhibition triggers Aβ release and, in turn, enhances glucose uptake. Answering this question is relevant to understanding whether Aβ monomers support activity-

For Review Only

dependent brain metabolism, a function that could be compromised in AD and other disorders characterized by PFC hypometabolism.

Materials and Methods

Primary neuronal cultures: preparation and treatments

Animal care and experimentation were in accordance with national and institutional guidelines. Cultures of pure cortical neurons were obtained from rats at embryonic day 15 and grown as described previously[5]. Cortical cells were plated onto 24-well plates pre-coated with 0.1 mg mL^{-1} poly-D-lysine (Sigma-Aldrich, Cat. No. P6407) and incubated at 37 °C with 5% CO_2 in a humidified atmosphere. Cytosine arabinoside (Ara-C, 5 μM) (Sigma Aldrich, Cat. No. C1768) was added to the cultures 18 h after plating to avoid the proliferation of non-neuronal elements and was kept for 3 days before partial medium replacement. Experiments were performed in mature culture at 14 days in vitro (DIV). All experiments were performed in artificial excitable CSF (aCSF) (3.5 mM KCl, 126 mM NaCl, 1.25 mM NaH2PO4, 0.5 mM MgSO4, 1 mM CaCl2, 26 mM NaHCO3) containing 10 μM glycine and, when required, 3 mM glucose. To block Aβ release, 100 nM γ-secretase inhibitor IX (DAPT) (Sigma-Aldrich, Cat No. 565770) was added to the artificial excitable CSF 2 hrs before the experiments.

In vitro glucose and lactate assays

Glucose content in the culture buffer (aCSF) was measured by Cayman's Glucose Colorimetric Assay Kit (Cat. No. 10009582). Lactate release in the culture buffer was quantified by Sigma-Aldrich Lactate Assay Kit (Cat. No. MAK 570, colorimetric detection). In both cases, following the technical instructions, absorbance was read by a spectrophotometric multiwell plate reader.

Preparation of $A\beta_{42}$ monomers

For Review Only

$A\beta_{42}$ (HFIP-treated) was purchased from Bachem Distribution Services GmbH, Germany, dissolved at a 5 mM concentration in anhydrous dimethyl sulfoxide (DMSO) and stored at −20 °C. At the time of its use, a solution of 100 µM Aβ in ice-cold DMEM F-12 was prepared and allowed to oligomerize overnight at 4 °C. Monomers were isolated from the peptide suspension, containing both monomers and oligomers, by filtration through 10 kD cutoff filters as previously described[5].

Glucose uptake assessment in the mouse PFC

All experimental procedures were approved by the institutional animal care and use committee and authorized by the Italian Ministry of Health (authorization n. 149/2021-PR). Female 129/SVE mice (12 weeks old) were fasted for 16 hours prior to the experiment. When indicated, mice received an intraperitoneal (i.p.) injection of either the γ-secretase inhibitor IX, DAPT, (100 mg/kg) or the γ-secretase modulator JNJ-40418677 (80 mg/kg) (MCE, Cat. No. HY-100604), or an equal volume of vehicle, at the doses previously reported to effectively modulate γ-secretase activity[12-13]. Three hours later, animals were injected i.p. with memantine (20 mg/kg) (Sigma Aldrich, Cat. No. M9292) or saline; the memantine dose was selected based on previous studies demonstrating its central nervous system activity[14]. Fifteen minutes after memantine administration, mice received an i.p. injection of 2-DG (3 mg/kg) (Sigma Aldrich, Cat. No. D8375) at a dose known to allow reliable detection of glucose uptake in brain tissue[15]. Animals were euthanized 45 minutes after 2-DG injection. The PFC was rapidly dissected; one hemisphere was immediately processed for 2-deoxyglucose-6-phosphate (2-DG6P) assessment, while the contralateral hemisphere was stored at -80°C for subsequent Western blot analysis or ELISA quantification. For 2-DG6P assessment, the dissected tissue was homogenized in 10 mM TRIS HCl (pH 8.1) and subsequently heated at 95°C for 15 minutes. Following centrifugation (18,000 x *g* for 15 minutes at 4°C) to remove the cell debris, the resulting supernatants were used to measure 2-DG uptake. The measurement of 2-DG uptake in the tissue was performed using the 2-

For Review Only

DG uptake measurement kit (CSR-OKP-PMG-K01TE, Cosmo Bio, Tokyo, Japan) according to the manufacturer's instructions, enabling the 2-DG uptake into the cells to be quantified as the amount of 2-DG6P without the interference of the remaining extracellular 2-DG based on the enzymatic photometric method[16].

Western Blot analysis

PFC samples were homogenized in RIPA buffer supplemented with 1X protease (Sigma-Aldrich, Cat. No. P8340) and phosphatase inhibitors (Sigma-Aldrich, Cat. No. P5726 and P0044) using ULTRA-TURRAX homogenizer, then sonicated (three 10-second pulses on ice) and centrifuged at 14,000 x g for 15 minutes at 4°C. The supernatant was collected, and protein concentration was determined using Pierce™ BCA Protein Assay Kit (ThermoFisher Scientific, Cat. No 23227), measuring absorbance at 562 nm with SpectraMax® ABS Plus microplate reader (Molecular Devices, San Jose, CA, USA). Twenty mg of total protein per sample were separated on 10% or 4-12% Tris-glycine gels and transferred to PVDF membranes. Membranes were blocked in Intercept® (PBS) blocking buffer (LI-COR, Cat. No 927-70003) for 1 hour at room temperature, then incubated with anti-APP-C-terminal fragment (1:1000, BioLegend, Cat. No 802801) and anti-actin (1:1000, Merck, Cat. No A4700), as housekeeping, in blocking buffer at 4°C overnight. After washing in Tris-buffered saline (TBS) with 0.1% Tween 20, membranes were incubated with IRDye® 800CW goat anti-mouse IgG and 680LT goat anti-mouse IgG secondary antibodies (1:15000, LI-COR, Cat. No 926-32210 and 926-68070, respectively) for 1 hour at room temperature. Hybridization signals were detected using the Odyssey® Infrared Imaging System (LI-COR Biosciences, Lincoln, NE, USA).

For Review Only

Extraction of soluble Aβ_{42} and ELISA quantification

Frozen PFC samples (30-40 mg) were homogenized using disposable pestles in 10 volumes (w/v) of ice-cold Tris-buffered saline (TBS) supplemented with a protease inhibitor cocktail

(Sigma-Aldrich P8340). The homogenate was centrifuged at 20,000 x g for 30 minutes at 4°C. The resulting supernatant (TBS-soluble fraction) was carefully collected, and protein concentration was determined using the Pierce™ BCA Protein Assay Kit, measuring absorbance at 562 nm with SpectraMax® ABS Plus microplate reader. $A\beta_{42}$ levels in the same fraction were quantified using a commercial ELISA kit specific for mouse $A\beta_{42}$ (Invitrogen, Cat. No KMB3441) according to the manufacturer's instructions, and absorbance was measured at 450 nm using the same microplate reader. $A\beta_{42}$ concentrations were normalized to total protein content as picograms of $A\beta_{42}$ per microgram of sample protein.

Statistical analysis

Statistical significance was assessed by one-way analysis of variance (ANOVA), followed by post hoc Fisher's LSD or Student-Newman-Keuls test for multiple comparisons, as appropriate. For comparisons between two groups, a two-tailed Student's T test was used. All analyses were performed using SigmaPlot 12.5.

For Review Only

Results

Memantine recapitulates the metabolic effects of excitation in cortical neurons

We previously demonstrated that a brief neuronal excitation with kainate (100 μM) stimulates glucose uptake and aerobic glycolysis through a mechanism requiring endogenous Aβ production[4]. To test whether memantine, which increases neuronal activity by disinhibiting GABAergic interneurons[14], mimics this effect, we first examined its action on pure cortical neuronal cultures. Memantine (1, 4, and 10 μM) stimulated glucose uptake only at the highest concentration tested (data not shown). Specifically, following glucose deprivation and re-addition of 3 mM glucose, memantine (10 μM) significantly increased glucose consumption within 10 minutes (Figure 1A) and lactate release within 40 minutes (Figure

1B). Both effects were prevented by pre-incubation with the γ-secretase inhibitor IX, DAPT (100 nM), indicating that memantine promotes glucose uptake and aerobic glycolysis through a γ-secretase-dependent pathway, mirroring the effect of kainate-induced excitation.

Memantine increases glucose uptake and activates γ-secretase in the PFC *in vivo*

We next examined whether memantine enhances glucose uptake in the living brain. A single intraperitoneal injection of memantine (20 mg/kg) in 12-week-old female mice significantly increased 2-DG6P accumulation in the PFC compared to saline-injected controls (Figure 2A). To determine whether memantine activates γ-secretase in the PFC, we measured APP C-terminal fragment (APP-CTF) levels. Because APP-CTFs are direct substrates of γ-secretase, their accumulation indicates reduced γ-secretase activity, whereas decreased levels reflect enhanced γ-secretase cleavage[17]. Memantine treatment reduced APP-CTF levels compared to controls (Figure 2B), consistent with enhanced γ-secretase cleavage of APP.

Blockade of γ-secretase prevents memantine-induced cerebral glucose uptake

If memantine-induced glucose uptake depends on γ-secretase activity, pharmacological inhibition of the enzyme should abolish the effect. Pre-treatment with the γ-secretase inhibitor IX, DAPT (100 mg/kg), three hours before memantine challenge significantly reduced 2-DG6P accumulation in the PFC (Figure 3A). DAPT-treated mice showed increased APP-CTF levels compared to mice receiving memantine alone (Figure 3B), confirming effective γ-secretase blockade. These results demonstrate that memantine-induced cerebral glucose uptake requires active γ-secretase and, consequently, activity-dependent Aβ production.

Selective lowering of $A\beta_{42}$ by a γ-secretase modulator blunts the metabolic response to memantine

For Review Only

To determine whether the effect of memantine specifically requires the Aβ_{42} isoform, we used the γ-secretase modulator JNJ-40418677, which selectively reduces Aβ_{42} without affecting Notch processing[12]. Mice pre-treated with JNJ-40418677 (80 mg/kg) three hours before memantine showed a marked reduction in memantine-stimulated 2-DG6P accumulation in the PFC (Figure 4A). Under the same conditions, soluble Aβ_{42} levels in the PFC, measured by ELISA, were significantly reduced compared to control or memantine-alone groups (Figure 4B). These findings indicate that memantine-induced PFC glucose uptake depends on the availability of soluble Aβ_{42}, supporting a role for the monomeric peptide in activity-dependent metabolic coupling.

Discussion

The PFC is a region of high metabolic demand and is particularly vulnerable to hypometabolism in AD[8-9]. The PFC exhibits high baseline aerobic glycolysis, and the spatial distribution of this metabolic signature correlates with later Aβ deposition[10]. We therefore set out to investigate the mechanisms that support activity-dependent energy metabolism in this region. Female mice were used in this study as a first step toward future experiments in the CRND8 transgenic mouse model of AD[18]. In this model, females develop an aggressive pathology[19] and, in the future, will be used to investigate neurometabolic coupling across the progression of AD pathology, from the pre-plaque stage to plaque deposition within 16 weeks[18].

Importantly, the present finding that identifies Aβ_{42} as a physiological neurometabolic coupler in the PFC stands on its own as a discovery, independent of any future translational and clinical applications.

To probe activity-dependent metabolic coupling in the PFC, we needed a pharmacological tool that could reliably and transiently increase network activity. Memantine, an

For Review Only

uncompetitive NMDA receptor antagonist currently used for the treatment of AD, is known to disinhibit pyramidal neurons by preferentially blocking NMDA receptors on GABAergic interneurons[14]. We reasoned that memantine-induced disinhibition would increase presynaptic activity and, consequently, trigger activity-dependent Aβ release. We first tested *in vitro* whether memantine could stimulate glucose uptake and metabolism (i.e, aerobic glycolysis) in an Aβ-dependent manner, using pure cortical neuronal cultures. We tested memantine at 1, 4, and 10 μM; only the highest concentration was found to be effective. This concentration is commonly used *in vitro* to achieve NMDA receptor blockade[20] while preserving fast off-rate kinetics[21]. Using this concentration, memantine stimulated glucose uptake and lactate release in a γ-secretase-dependent manner, recapitulating our previous findings with kainate-induced depolarization in cultured neurons[4].

We then translated these observations to the living brain, showing that memantine administered systemically at a dose effective for brain activation (20 mg/kg)[14] increased 2-DG uptake in the mouse PFC and reduced APP C-terminal fragment levels, consistent with enhanced γ-secretase activity[17]. The finding that the γ-secretase inhibitor DAPT prevented memantine-induced glucose uptake both *in vitro* and *in vivo* directly links the metabolic response to ongoing Aβ production. To determine whether the effect specifically required the $Aβ_{42}$ isoform, we used the γ-secretase modulator JNJ-40418677, which selectively lowers $Aβ_{42}$ without affecting Notch processing[12]. Pre-treatment with JNJ-40418677 blunted memantine-induced glucose uptake in the PFC and concomitantly reduced soluble $Aβ_{42}$ levels, as measured by ELISA. The mean level of soluble $Aβ_{42}$ in control PFC was 0.011 pg/μg protein, which falls within the low picogram range expected for endogenous Aβ in wild-type mouse brain[22]. The observation that JNJ-40418677, which reduces $Aβ_{42}$ without altering total Aβ or Notch signaling, phenocopied the effect of DAPT strongly implicates the monomeric $Aβ_{42}$ as the critical effector of memantine-induced metabolic activation. A limitation of our *in vitro* experiments is that we did not directly measure Aβ release following

For Review Only

memantine challenge. However, and more important, we addressed the issue *in vivo* by quantifying soluble $A\beta_{42}$ levels in the PFC and showing that they are reduced under conditions where the metabolic response is blunted.

For the *in vivo* experiments, we used a non-radioactive enzymatic method for 2-DG6P quantification that is sensitive and reliable[16] but requires rapid tissue processing. The time-critical nature of the procedure necessarily limits the number of animals that can be processed per experimental session. Consequently, determinations were collected across multiple independent sessions, and the consistency of the observed effects supports the robustness of the findings despite constraints on group size. The 2-DG method measures total tissue glucose uptake, encompassing both neuronal and glial contributions. However, the prevention of memantine-induced glucose uptake by γ-secretase inhibitors points to acute, activity-dependent Aβ production. Although glial cells can produce Aβ - primarily as N-terminally truncated species[23] - and are sensitive to γ-secretase inhibitors[24-25], their Aβ production typically occurs in response to pathological or inflammatory stimuli and unfolds over hours rather than minutes[25]. Therefore, the rapid (minutes) time course of the memantine challenge favors activity-dependent neuronal release. This conclusion is reinforced by our experiments in pure neuronal cultures, in which the same γ-secretase-dependent response to memantine occurs without any glial contribution. The demonstration that memantine-induced PFC glucose uptake requires soluble $A\beta_{42}$ raises the question of whether distinct pathological conditions - including AD[8-9], late-life depression[26], frontotemporal dementia[27], and normal aging[28] - converge on failure of this Aβ-dependent metabolic coupling. If so, the consequence could be a common inability to adequately fuel PFC neurons during periods of high demand, suggesting that impairment of this pathway might represent a shared endpoint across multiple disorders. The current paradigm for activity-dependent metabolic support is the astrocyte-neuron lactate shuttle (ANLS), in which astrocytic glycolysis produces lactate that is shuttled to neurons to fuel oxidative

For Review Only

phosphorylation[29]. The mechanism we describe is fundamentally different: it is neuron-autonomous, rapid, and triggered by presynaptic Aβ release following increased network activity. Rather than replacing the ANLS, this intrinsic system may complement it by providing immediate local energy support during sustained firing before glial responses are fully engaged.

Acknowledgements

We thank the animal care and technical staff at CAPIR (Center for Advanced Preclinical In Vivo Research), University of Catania, for their assistance.

Funding

This work was supported by discretional research funds available to A. Copani

Competing interests

The authors report no competing interests.

Data availability

Data are available from the corresponding author upon request.

For Review Only

References

1. Selkoe DJ. Physiological production of the β-amyloid protein and the mechanism of Alzheimer's disease. *Trends Neurosci*. 1993;16(10): 403-409.
2. Copani A. The underexplored question of β-amyloid monomers. *Eur J Pharmacol.* 2017; 817: 71-75.
3. Giuffrida ML, Tomasello MF, Pandini G, et al. Monomeric ß-amyloid interacts with type-1 insulin-like growth factor receptors to provide energy supply to neurons. Front Cell Neurosci. 2015; 9:297.
4. Santangelo R, Giuffrida ML, Satriano C, et al. β-amyloid monomers drive up neuronal aerobic glycolysis in response to energy stressors. *Aging* 2021; 13(14):18033-18050.
5. Giuffrida ML, Caraci F, Pignataro B, et al. Beta-amyloid monomers are neuroprotective. *J Neurosci.* 2009; 29(34): 10582-7.

6. Espay AJ, Ezzat K, Kepp KP, et al. Restoring amyloid-β42 and γ-secretase function in Alzheimer's disease. *Brain*. 2025;148(11): 3856-3864.
7. Abanto J, Dwivedi AK, Imbimbo BP, Espay AJ. Increases in amyloid-β42 slow cognitive and clinical decline in Alzheimer's disease trials. *Brain*. 2024;147(10): 3513-3521.
8. Fouquet M, Desgranges B, Landeau B, et al. Longitudinal brain metabolic changes from amnestic mild cognitive impairment to Alzheimer's disease. *Brain.* 2009;132 (Pt 8): 2058-67.
9. Klupp E, Grimmer T, Tahmasian M, et al. Prefrontal hypometabolism in Alzheimer disease is related to longitudinal amyloid accumulation in remote brain regions. *J Nucl Med.* 2015; 56(3): 399-404.
10. Vlassenko AG, Vaishnavi SN, Couture L, et al. Spatial correlation between brain aerobic glycolysis and amyloid-β (Aβ) deposition. *Proc Nation Acad of Sci (USA)* 2010;107(41): 17763-7.
11. Povysheva NV, Johnson JW. Effects of memantine on the excitation-inhibition balance in prefrontal cortex. *Neurobiol Dis*. 2016; 96:75-83.
12. Van Broeck B, Chen JM, Tréton G, et al. Chronic treatment with a novel γ-secretase modulator, JNJ-40418677, inhibits amyloid plaque formation in a mouse model of Alzheimer's disease. *Br J Pharmacol*. 2011;163(2):375-89.
13. Lanz TA, Himes CS, Pallante G, et al. The γ-secretase inhibitor N-[N-(3,5-difluorophenacetyl)-l-alanyl]-S-phenylglycine t-butyl ester reduces Aβ levels in vivo in plasma and cerebrospinal fluid in young (plaque-free) and aged (plaque-bearing) Tg2576 mice. *Journal of Pharmacology and Experimental Therapeutics*. 2003;305(3):864-871.
14. Dedeurwaerdere S, Wintmolders C, Straetemans R, et al. Memantine-induced brain activation as a model for the rapid screening of potential novel antipsychotic compounds: exemplified by activity of an mGlu2/3 receptor agonist. Psychopharmacology (Berl). 2011; 214(2):505-14.
15. Hiyoshi T, Marumo T, Hikichi H, et al. Neurophysiologic and antipsychotic profiles of TASP0433864, a novel positive allosteric modulator of metabotropic glutamate 2 receptor. *J Pharmacol Exp Ther*. 2014; 351(3):642-53.
16. Saito K, Lee S, Shiuchi T, Toda C, et al. An enzymatic photometric assay for 2-deoxyglucose uptake in insulin-responsive tissues and 3T3-L1 adipocytes. *Anal Biochem*. 2011; 412: 9-17.
17. Sharples RA, et al. Inhibition of gamma-secretase causes increased secretion of amyloid precursor protein C-terminal fragments in association with exosomes. *FASEB J*. 2008; 22(5):1469-1478.
18. Chishti MA, Yang DS, Janus C, Phinney AL, et al. Early-onset amyloid deposition and cognitive deficits in transgenic mice expressing a double mutant form of amyloid precursor protein 695. *J Biol Chem*. 2001; 276(24): 21562-70.
19. Keyvani K, Münster Y, Kurapati NK, et al. Higher levels of kallikrein-8 in female brain may increase the risk for Alzheimer's disease. *Brain Pathology*. 2018; 28(6): 947-964.
20. Frankiewicz T, Parsons CG. Memantine restores long term potentiation impaired by tonic N-methyl-D-aspartate (NMDA) receptor activation following reduction of Mg2+ in hippocampal slices. *Neuropharmacology*. 1999; 38(9):1253-1259.
21. Kotermanski SE, Johnson JW. Potency, voltage-dependency, agonist concentration-dependency, blocking kinetics and partial untrapping of the uncompetitive N-methyl-

For Review Only

D-aspartate (NMDA) channel blocker memantine at human NMDA (GluN1/GluN2A) receptors. *Neuropharmacology*. 2009; 56(5): 866-875.

22. Peng HB, de Lannoy IAM, Pang KS. Measuring Amyloid-β Peptide Concentrations in Murine Brain with Improved ELISA Assay. *Current Protocols*. 2021; 1: e253.
23. Oberstein TJ, Spitzer P, Klafki HW, et al. Astrocytes and microglia but not neurons preferentially generate N-terminally truncated Aβ peptides. *Neurobiol Dis.* 2015; 73:24-35.
24. Sozio P, Rapino M, Di Valerio V, et al. pPKCα-mediated effect on in vitro Aβ production in response to gamma secretase inhibitor LY411575 in rat CTXTNA2 astrocytes. *J Biol Regul Homeost Agents*. 2012; 26(2): 245-51.
25. Sharifulina S, Khaitin A, Guzenko V, et al. Expression of Amyloid Precursor Protein, Caveolin-1, Alpha-, Beta-, and Gamma-Secretases in Penumbra Cells after Photothrombotic Stroke and Evaluation of Neuroprotective Effect of Secretase and Caveolin-1 Inhibitors. *Biomedicines*. 2022;10(10):2655.
26. Ferrando R, Hernandes D, Ghini BG, Coutinho AM. PET Imaging in Psychiatric Disorders. *Seminars in Nuclear Medicine*. 2025; 55(4): 587-604.
27. Tuma RLE, Coutinho AM, Carneiro CG, et al. Quantifying metabolic decrement in brain FDG-PET of a complex frontotemporal dementia case. *Dementia & Neuropsychologia*. 2025;19: e20240277.
28. Chételat G, Landeau B, Salmon E, et al. Relationships between brain metabolism decrease in normal aging and changes in structural and functional connectivity. *Neuroimage*. 2013;76: 167-77.
29. Pellerin L, Magistretti PJ. Glutamate uptake into astrocytes stimulates aerobic glycolysis: a mechanism coupling neuronal activity to glucose utilization. *Proc Nat Acad Sci (USA)*. 1994; 91(22):10625-10629.

For Review Only

Figure legends

Figure 1. Memantine promotes glucose uptake and lactate release in cultured rat cortical neurons via a γ-secretase-dependent pathway. Following 2 hours of glucose deprivation, 3 mM glucose was added to neuronal cultures. A treatment with memantine 10 μM) stimulated glucose uptake after 10 min (A) and lactate release after 40 min (B). Glucose consumption was measured as glucose (mg/dl) remaining in the incubation buffer 10 minutes following re-addition. With respect to the initial 3 mM glucose concentration, no glucose uptake occurred within 10 min unless memantine was added. The γ-secretase inhibitor IX (DAPT, 100 nM) prevented memantine-stimulated glucose uptake (A) and lactate release at 40 min (B). Bars represent the means $\pm$ SEM of 4 determinations. In A, $p< 0.001$ vs *control (CTRL) or **memantine alone. In B, $p< 0.001$ vs *CTRL or $p < 0.05$ vs **memantine alone; one-way ANOVA with post hoc Fisher LSD multiple comparison method.

Figure 2. Memantine enhances glucose uptake and stimulates γ-secretase activity in the mouse PFC. A) Increased PFC glucose uptake after memantine. A single injection of memantine led to significantly higher 2-DG6P levels in 12-week-old female SVE mice within 60 minutes. Bars represent means $\pm$ SEM of 9-11 determinations from 5 experiments. Within each experiment, the mean value of the saline condition was set to 100%, and each individual data point was normalized accordingly; these normalized values were then pooled across experiments, so that data are expressed as percentage of 2-DG6P accumulation relative to saline. Basal levels of 2-DG6P in the PFC under control conditions (saline-injected mice) ranged from 0.05 to 3.7 pmol/μg protein across experiments, reflecting the inherent variability of *in vivo* measurements. *$P < 0.05$ vs saline by two-tailed t test. B) Western blot analysis of PFC lysates from memantine-treated mice showed reduced levels of APP C-terminal fragments (APP-CTF), indicating enhanced γ-secretase activity. Holo APP = full

For Review Only

length APP. The 42 kD band of β-actin is shown *as a* loading control. The slight increase in protein loading in the memantine-treated sample explains the corresponding increase in holo-APP levels. Nevertheless, APP-CTF levels were reduced after memantine treatment.

Figure 3. Inhibition of γ-secretase by DAPT reduces memantine-induced glucose uptake in the mouse PFC. A) Pre-treatment with DAPT reduced glucose uptake in the mouse PFC following a memantine challenge. Bars represent means ± SEM of 6-7 determinations from 3 experiments. Within each experiment, the mean value of the memantine-treated condition was set to 100%, and each individual data point was normalized accordingly. These normalized values from all experiments were then pooled, so that data are expressed as percentage of 2-DG6P accumulation relative to memantine. **P* < 0.05 vs memantine alone by two-tailed t test. B) Western blot analysis of PFC lysates from DAPT-pretreated, memantine-injected mice showed increased levels of APP C-terminal fragments (APP-CTF), indicating reduced γ-secretase activity. Holo APP = full length APP.

Figure 4. The selective Aβ42-lowering γ-secretase modulator, JNJ 40418677, reduces memantine-induced cerebral glucose uptake in the mouse PFC

A) Pre-treatment with JNJ 40418677 reduced glucose uptake in the mouse PFC following a memantine challenge. Bars represent means ± SEM of 9 determinations from 3 experiments. Within each experiment, the mean value of the memantine-treated condition was set to 100%, and each individual data point was normalized accordingly. These normalized values from all experiments were then pooled, so that data are expressed as percentage of 2-DG6P accumulation relative to memantine. **P* < 0.001 vs memantine alone by two-tailed t test.

For Review Only

B) Soluble $A\beta_{42}$ levels were reduced in the PFC of JNJ 40418677-pretreated, memantine-injected mice, as assessed by ELISA. The mean level of soluble $A\beta_{42}$ in control (saline-injected) mice was 0.011 pg/µg protein. Bars represent means $\pm$ SEM of 6-7 determinations from two experiments. Within each experiment, the mean value of the control condition was set to 100%, and each individual data point was normalized accordingly; these normalized values were then pooled across experiments, so that data are expressed as percentage of soluble $A\beta_{42}$ relative to control. $P < 0.05$ vs *control (CTRL) or **memantine alone; one-way ANOVA with post hoc Student-Newman-Keuls for multiple comparison method.

For Review Only

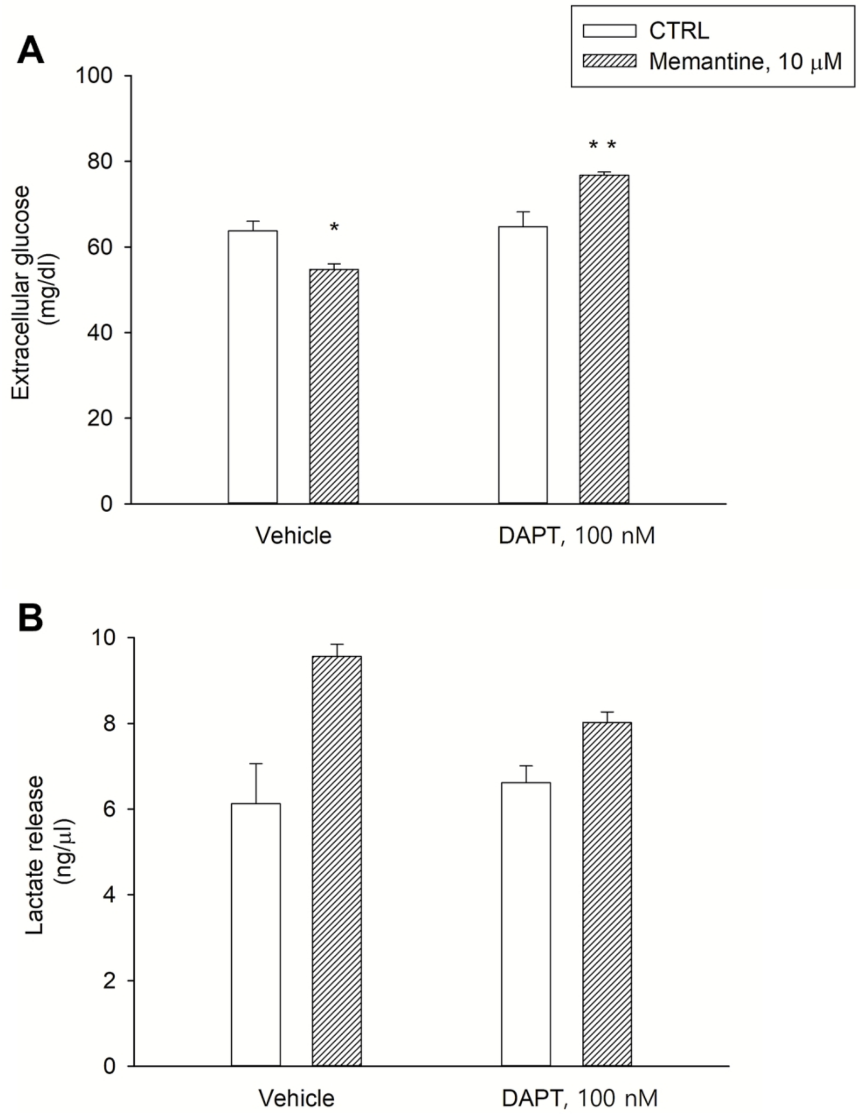

Figure 1

130x176mm (300 x 300 DPI)

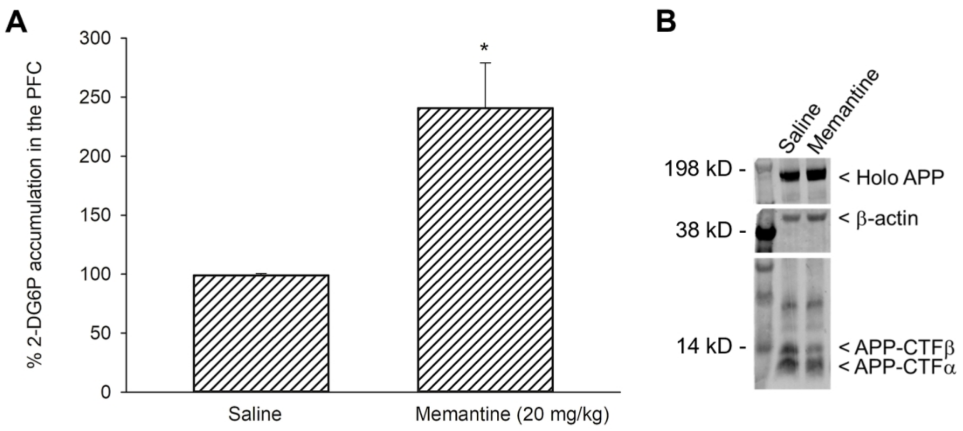

Figure 2

159x78mm (300 x 300 DPI)

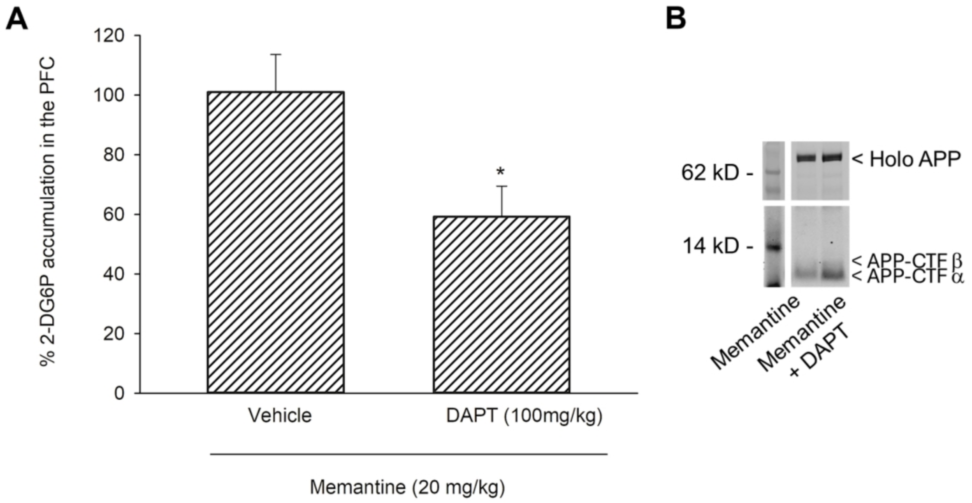

Figure 3

157x94mm (300 x 300 DPI)

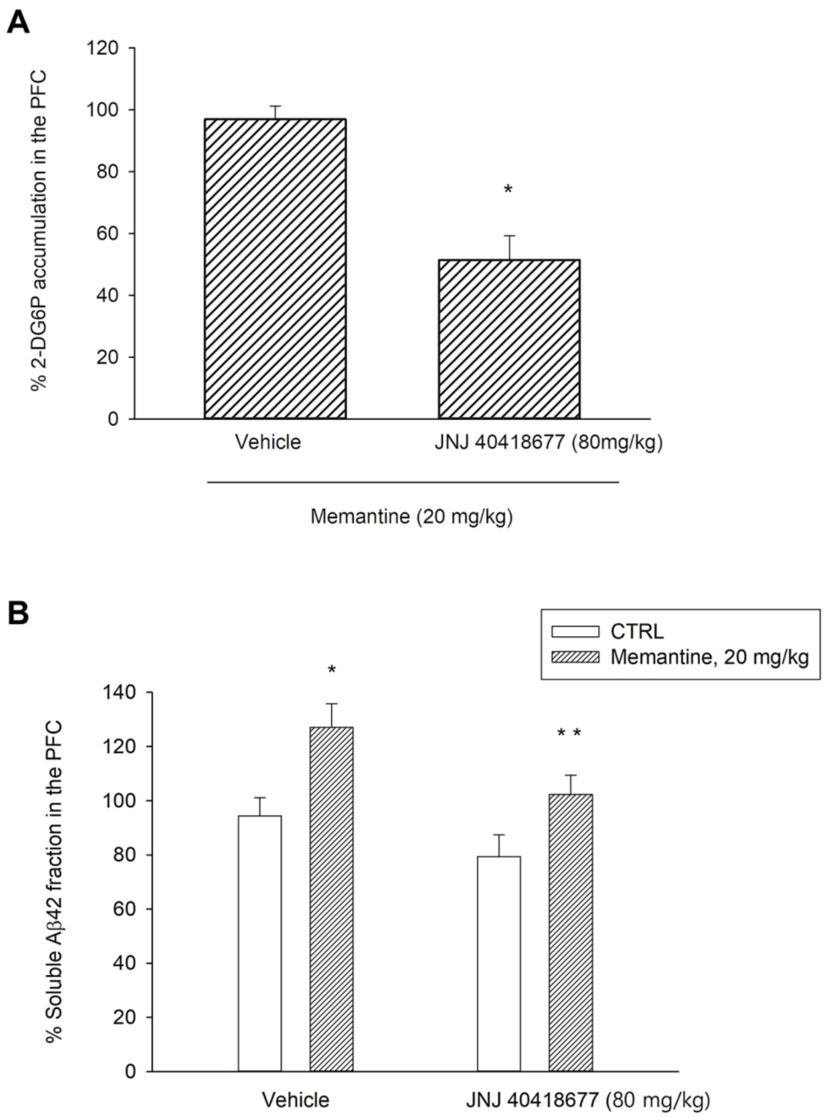

Figure 4

131x182mm (300 x 300 DPI)

Full images of Figure 2B WB

Odyssey Image

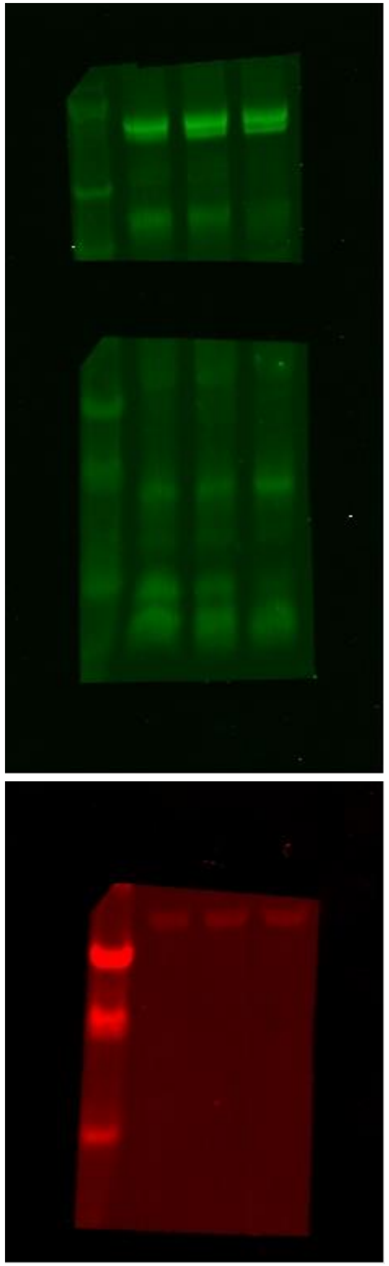

The membrane was cut into two parts for antibody incubation.

Grey Level Image

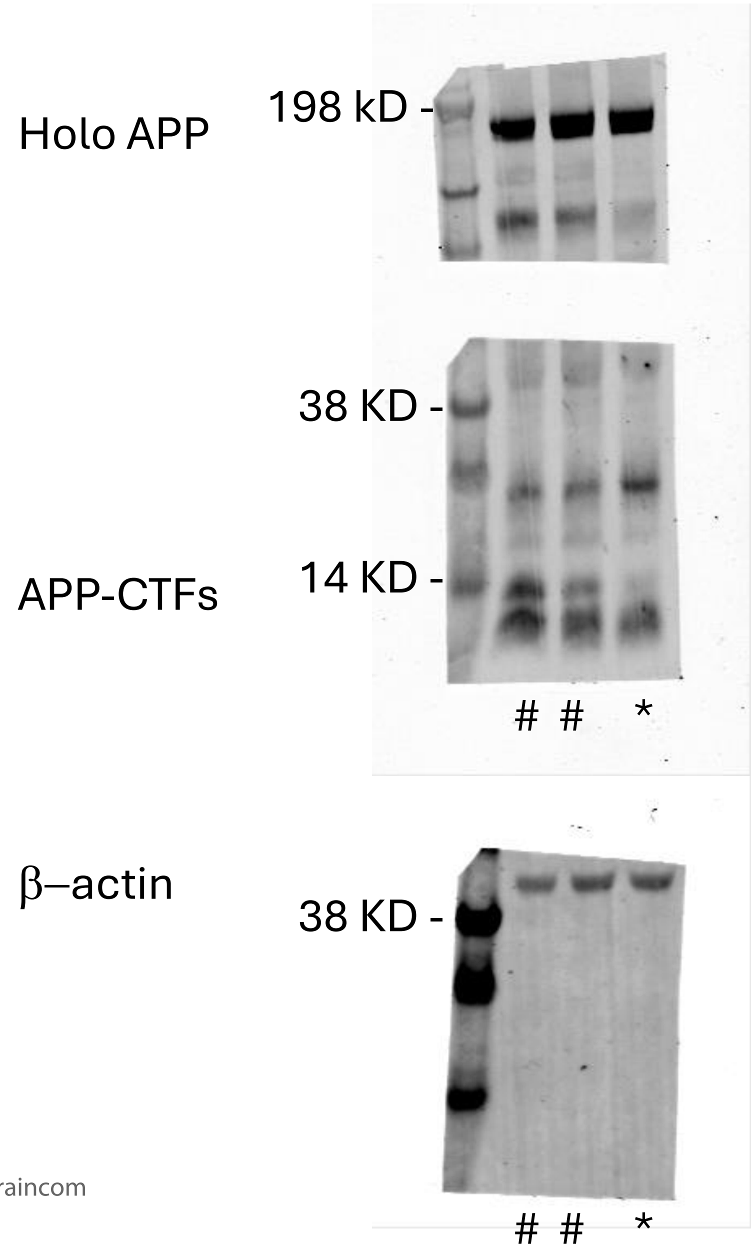

Shown in the manuscript
* Not relevant to this study

Full images of Figure 3B WB

Odyssey Images

Grey level Images

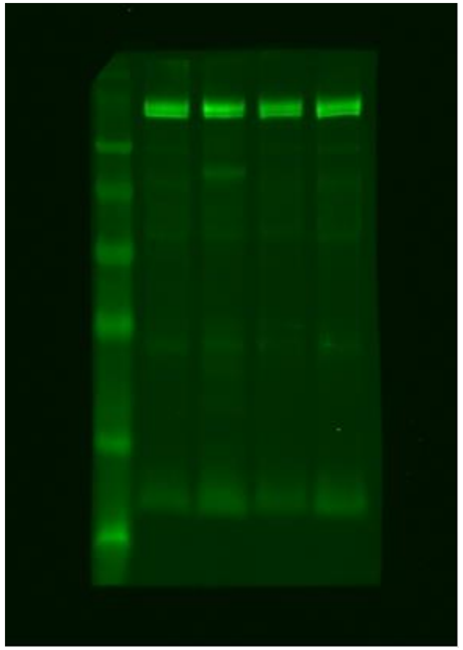

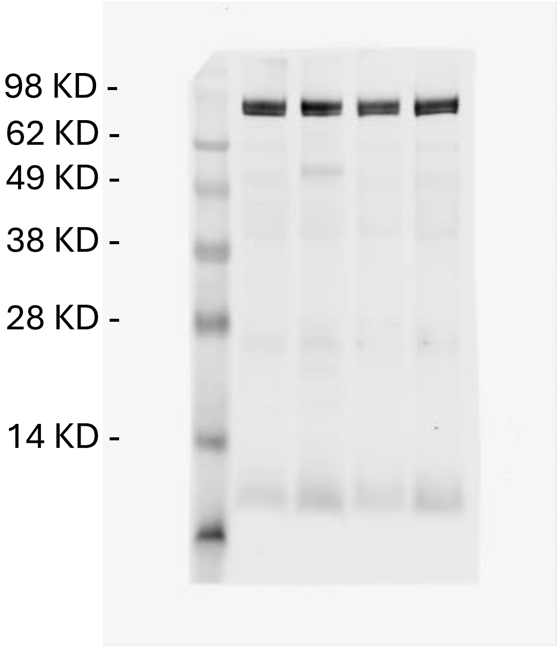

The membrane was cut at 28 kD and re-blotted with primary and secondary antibodies to improve the signal below 28 KD

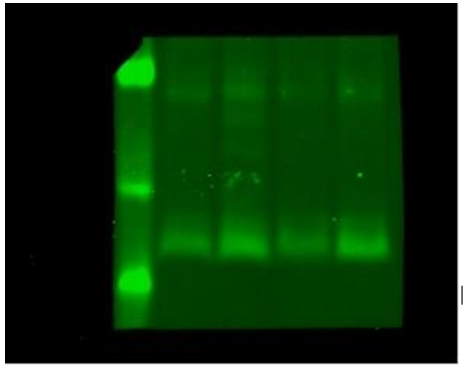

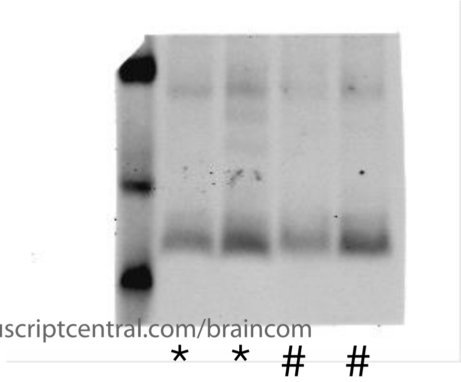

* Not relevant to this study
# Shown in the manuscript
