# Supplementary material for: Activity-dependent glucose uptake in the mouse prefrontal cortex depends on endogenous beta-amyloid production
Source: Brain Commun. 2026 Oct 28;8(6):fcag412. doi: 10.1093/braincomms/fcag412 (PMC13739058; doi:10.1093/braincomms/fcag412)

UNIVERSITÀ degli STUDI di CATANIA

DIPARTIMENTO di SCIENZE del FARMACO e della SALUTE

Catania, 23 July 2026

Dear Dr. Higuera-Matas and Prof. Belin,

We thank the editors and reviewers for their thoughtful and constructive comments on our manuscript BRAINCOM-2026-768 entitled "Activity-dependent glucose uptake in the mouse prefrontal cortex depends on endogenous Aβ production." We are grateful for the recognition that our study is conceptually novel and well executed, and we have carefully addressed all the concerns raised.

We have revised the manuscript in accordance with all the reviewers' suggestions and the editorial requirements. All changes are highlighted in **red** in the revised manuscript. A point-by-point response to each comment is provided below.

Furthermore, I would like to bring to the attention of the Editors and Reviewers that a new author, Dr. Rosa Santangelo, has been added. This addition is due to the fact that Dr. Santangelo's name was inadvertently omitted from the original submission, whereas it should have been included, as Dr. Santangelo performed the 2-DG uptake assay in the mouse prefrontal cortex.

Responses to Reviewer 1

Comment: “The study is well executed and the findings relevant. I suggest the authors clarify in the abstract and discussion the major clinical implication: these findings argue for moving gamma-secretase modulation efforts from lowering to increasing gamma-secretase activity and Aβ42 levels. This is more important than the current conclusion, namely that the physiological function of Aβ42 “may be compromised in AD and other disorders characterized by PFC hypometabolism.” This, in fact, may be included in the discussion but is not the most critical implication”

Response: We thank the reviewer for this important suggestion. We agree that the clinical implications of our findings should be more prominently emphasized. We have revised the **abstract (lines 31-34)** to state that our findings " have direct clinical implications for AD: they argue against therapeutic strategies aimed at lowering Aβ42 levels, challenging the paradigm of simply reducing Aβ burden”. In the **discussion (lines 324-332)**, we have expanded the clinical implications to emphasize that our results support a loss-of-function hypothesis and caution against Aβ42-lowering strategies.

Comment: “Consider adding the specific levels of Aβ42 in the appropriate figures (e.g. Fig 4) and in the corresponding text.”

Response: We agree. We have added the specific Aβ42 levels in the **Results section (lines 223-225)**, stating that " The absolute level in control mice was 0.011 pg/μg protein, and the normalized data show that memantine increased soluble Aβ42 relative to control, an effect prevented by JNJ-40418677 (Figure 4B).

Comment: "Lastly, consider noting in the discussion that these results support a loss of function hypothesis for Alzheimer's disease where the loss of functional, soluble Aβ42 (both through an endopeptidase, neprilysin, and as it aggregates into amyloid) may be more detrimental to the brain than the corresponding accrual of insoluble amyloid (PMID: 38270185)".
Response: This comment is closely related to the first point and has been addressed in the same section of the **discussion (lines 324-332)**. We now explicitly state that our findings "are consistent with a loss-of-function hypothesis for Alzheimer's disease, wherein the loss of functional, soluble Aβ42—whether through aggregation into amyloid or through degradation by endopeptidases such as neprilysin—could contribute to neuronal dysfunction independently of, or in addition to, the accumulation of insoluble amyloid (Espay et al., 2023)."

Response to Reviewer 2

Comment: "First, the manuscript does not directly demonstrate that memantine increases endogenous Aβ42 production or release. This is a key assumption of the proposed model, yet the conclusion is inferred from γ-secretase inhibition and modulation rather than from direct measurement of Aβ42 following memantine treatment. Demonstrating such an increase, or alternatively moderating the conclusions, would substantially strengthen the manuscript"

Response: We appreciate this concern and apologize for not being clear. We acknowledge that we did not measure Aβ42 release in vitro following memantine challenge. However, we did directly measure soluble Aβ42 levels in vivo following memantine challenge. As shown in **Figure 4B** and described in the **Results section (lines 223-225)** and **Discussion (lines 278-282)**, memantine increased soluble Aβ42 levels in the PFC, and pre-treatment with the selective Aβ42-lowering modulator JNJ-40418677 prevented this increase. Under the same experimental conditions, JNJ-40418677 also blunted memantine-induced glucose uptake (Figure 4A), providing a direct link between Aβ42 production and the metabolic response. Thus, the reviewer's concern is addressed by existing data, which we have now made more explicit in the revised manuscript and in the abstract.

Comment: "Second, APP-CTF levels are interpreted as evidence of altered γ-secretase activity. Although this is a reasonable surrogate marker, APP-CTF abundance may also be influenced by additional mechanisms affecting APP processing or turnover. This limitation should be acknowledged".

Response: We agree and have acknowledged this limitation in the Discussion (**lines 261-264**), where we now state: "While APP-CTF levels are a reasonable surrogate marker of γ-secretase activity, we acknowledge that their abundance may also be influenced by additional mechanisms affecting APP processing or turnover. Nevertheless, the consistency of the APP-CTF changes with the functional outcomes supports our interpretation."

Comment: "Third, the mechanistic conclusions rely exclusively on pharmacological approaches. While the use of DAPT and JNJ-40418677 is appropriate, pharmacological manipulation alone cannot unequivocally establish causality. This limitation should also be discussed".

Response: We have addressed this in the **Discussion (lines 288-290)**, where we now state: "We also recognize that our mechanistic conclusions rely exclusively on pharmacological approaches.

While the use of DAPT and JNJ-40418677 is appropriate, pharmacological manipulation alone cannot unequivocally establish causality. Genetic approaches, such as conditional APP knockout, would provide complementary evidence in future studies."

Comment: “Finally, glucose uptake is quantified in whole prefrontal cortex tissue. Since the assay cannot distinguish neuronal from glial glucose utilization, I believe statements suggesting a neuron-autonomous mechanism are stronger than warranted. A more balanced discussion acknowledging possible contributions from astrocytes and other brain cells would improve the manuscript.”

Response: We acknowledge that this limitation was already discussed in the original submission. However, we have now rephrased this section to be more balanced (**lines 317-323**). We now state: "The mechanism we describe appears to be largely neuron-autonomous and is triggered by presynaptic Aβ release following increased network activity. Rather than replacing the ANLS, this intrinsic system may complement it by providing rapid local energy support during sustained firing, potentially before glial responses are fully engaged. However, we acknowledge that the 2-DG method measures total tissue glucose uptake, and we cannot exclude the possibility of additional glial contributions, particularly at later time points”.

Response to Scientific Editor Team

We have complied with all the requirements from the Scientific Editor Team.

We have completed the ARRIVE 2.0 checklist and uploaded it as a supplementary file, as required for our in vivo study. The Methods Section has been implemented to fulfill the guideline checklist (red text).

We are confident that the revised manuscript now addresses all the concerns raised and meets the journal's standards. We thank the editors and reviewers once again for their time and valuable feedback, and we look forward to your favorable decision.

Sincerely,

Prof. Agata Copani, MD, PhD
Department of Drug and Health Sciences
University of Catania
Viale Andrea Doria 6
95125 Catania, Italy
Email: acopani@katamail.it

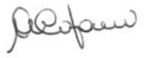

Supplement: Supplementary file 1 [file fcag412_supplementary_data.zip › Original_submission_Response_to_reviewers_comments.pdf]
