# Supplementary material for: Activity-dependent glucose uptake in the mouse prefrontal cortex depends on endogenous beta-amyloid production
Source: Brain Commun. 2026 Oct 28;8(6):fcag412. doi: 10.1093/braincomms/fcag412 (PMC13739058; doi:10.1093/braincomms/fcag412)

UNIVERSITÀ degli STUDI di CATANIA

DIPARTIMENTO
di SCIENZE del FARMACO e della SALUTE

Catania, 10 September 2026

Dear Dr. Higuera-Matas and Prof. Belin,

We thank you very much for the provisional acceptance of our manuscript BRAINCOM-2026-768 R1 entitled "Activity-dependent glucose uptake in the mouse prefrontal cortex depends on endogenous Aβ production." We sincerely appreciate the opportunity to publish our work in your journal and are grateful for the constructive feedback provided throughout the review process.

We confirm that we have carefully addressed all the minor points raised by the Reviewer and the Scientific Editor, including full compliance with your policies on data analysis and presentation. A detailed point-by-point response to each comment is provided below.

We hope that the revised version now fully meets the journal's requirements and look forward to your final decision.

All changes are highlighted in red in the revised manuscript.

Response to Reviewer 1

Comment: Please delete the word "simply" from the abstract and discussion. For instance, "...challenging the paradigm of simply reducing Aβ burden" implies that reducing Aβ burden ALONE is not sufficient, as it must be COMBINED with something else. This is inaccurate. A minor but more accurate conclusion is "This work establishes Aβ42 as a physiological neurometabolic coupler in the PFC and has direct clinical implications for AD: it supports therapeutic strategies aimed at preserving or restoring physiological Aβ42 levels rather than further reducing them".

Response: We thank the reviewer for his suggestion. The abstract has been modified accordingly.

Responses to the Scientific editor

Comment: Thank you for including a graphical abstract; however, we have one final minor request. Please clarify whether any external software was used (e.g. BioRender, etc.) to generate the graphics within the abstract. If BioRender was used, for instance, please include an acknowledgement at the bottom of the graphical abstract ("created using BioRender"), accompanied by a link to where the graphics can be found on the BioRender website.

Response: No external graphical software (e.g., BioRender) was used to generate the graphical abstract. All elements were created manually in Microsoft PowerPoint. The mouse silhouette, syringe, mouse brain, and scissors were hand-drawn in PowerPoint based on AI-generated reference outlines (ChatGPT), while all remaining graphical components were drawn entirely by hand. This has now been specified in the summary of the graphical abstract.

Comment: Please clarify whether any code was generated for the purpose of statistical analysis (e.g. using programs such as R, MATLAB, Python or otherwise). Please note that, if any analysis code was generated, we strongly encourage authors to make such code available to readers, either via online repository such as GitHub or attached directly as supplementary materials to the main manuscript.

Response: No code was generated for the statistical analysis. All statistical analyses were performed using SigmaPlot (Systat Software, Inc.), a point-and-click graphical statistics package that does not require or generate any scripting or programming code. No R, MATLAB, Python, or other programming-based tools were used at any stage of the analysis. Accordingly, there is no analysis code to deposit in a repository or provide as supplementary material.

Comments:

- Figures 1 and 3: please include a statement in the figure legend to indicate what each datapoint/dot in the bar chart represents (e.g. "each datapoint represents an independent measure from a single experiment/passage/determination" or otherwise).

- Figures 2A and 3A: please note that, where $N < 10$ for any group, we request that individual datapoints be shown in plots. Please show data points overlaid atop the bar charts in a similar manner to that of Figure 1. When including datapoints, please also state what each point represents in the figure legend. Finally, please spell out remaining abbreviations in the figure legend, such as "PFC".

Response: We have now addressed all the points raised and have applied the requested changes consistently across all figures in the manuscript.

Comment: Figures 2B and 3B: please note that we require full, uncropped images of gels to also be included as supplementary material. Please include the uncropped version of these gels as supplementary figures.

Response: full, uncropped images of the gels shown in Figures 2B and 3B were already provided to the reviewers during the peer-review process. We will now upload these uncropped gel images as Supplementary Figure accompanying the revised manuscript. We hope this satisfies the journal's requirements for full image disclosure.

Sincerely,

Prof. Agata Copani, MD, PhD
Department of Drug and Health Sciences
University of Catania
Viale Andrea Doria 6
95125 Catania, Italy
Email: acopani@katamail.it

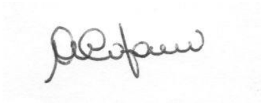

Supplement: Supplementary file 1 [file fcag412_supplementary_data.zip › Revision_1_ Response_to_reviewers_comments.pdf]
