# Supplementary material for: Activity-dependent glucose uptake in the mouse prefrontal cortex depends on endogenous beta-amyloid production
Source: Brain Commun. 2026 Oct 28;8(6):fcag412. doi: 10.1093/braincomms/fcag412 (PMC13739058; doi:10.1093/braincomms/fcag412)
Supplement: Supplementary file 1 [file fcag412_supplementary_data.zip › Revision_1_decision_letter.pdf]

From: brcoms.editorialoffice@paeditorial.co.uk
To:
CC:
BCC:
Subject: Brain Communications - Decision on Manuscript ID BRAINCOM-2026-768.R1
Body: 09-Sep-2026

Dear Prof. COPANI,

Thank you for your recent submission BRAINCOM-2026-768.R1
entitled "Activity-dependent glucose uptake in the mouse prefrontal cortex depends on endogenous Aβ production" to Brain Communications.

I am pleased to let you know that we would like to provisionally accept your manuscript entitled "Activity-dependent glucose uptake in the mouse prefrontal cortex depends on endogenous Aβ production" for publication in Brain Communications. There are still some minor points for you to address before final acceptance, but further peer-review will not be necessary. However, please note that we will not accept the paper unless you comply with our policies on data analysis and presentation as noted in the comments from the Scientific Editor.

Please respond to the comments shown below from reviewers and/or our Scientific Editor and submit a revised version.

Please format your revised manuscript according to our Instructions to Authors
https://academic.oup.com/braincomms/pages/General_Instructions
A summary of key points is attached to help with this. Please note that there can be substantial delays to publication if you don't resubmit in line with our requirements.

REMINDER: if you have not already done so, when submitting your revised manuscript, please include a short alternative-text description under the figure legend for each figure. Alt-text is required to ensure the accessibility of our publications. Guidance is available on the journal's Author Guidelines webpage and at https://academic.oup.com/pages/for-authors/journals/preparing-and-submitting-your-manuscript

To revise your manuscript, click the link below or log into https://mc.manuscriptcentral.com/braincom and enter your Author Centre, where you will find your manuscript title listed under "Manuscripts with Decisions." Under "Actions," click on "Create a Revision." Your manuscript number will be appended to denote a revision.

*** PLEASE NOTE: This is a two-step process. After clicking on the link, you will be directed to a webpage to confirm. ***

https://mc.manuscriptcentral.com/braincom?URL_MASK=6d48ff1254ab4460a8e01b0a2f1d5b7f

Please upload a revised manuscript with the changes you have made shown either with "track changes" or in coloured text.

When submitting your revised manuscript, you will be asked to respond to the reviewer(s) and editor's comments. Please be as specific as possible in your response to all of the comments.

The editorial team at Brain Communications also aims to increase transparency in scientific publishing and to demonstrate the value that peer review brings to the scientific process. Please therefore consider whether you are willing to have reviewer comments on your paper and your response published as supplementary data with the paper if it is accepted. This is entirely optional and will only be done if both the authors and reviewers agree. When submitting your revision you will have the opportunity to update the answer you entered previously.

IMPORTANT: Your original files are available to you when you upload your revised manuscript. Please delete any redundant files before completing the submission.

Because we are trying to facilitate timely publication of manuscripts submitted to Brain Communications, we would appreciate your revised manuscript as soon as possible. If it is not possible for you to submit your revision within 3 months, the option to submit a revision will expire, and we will consider your revised paper as a new submission. However, if you need a little more time, please get in touch and we can extend this deadline for you.

Please note that Brain Communications is a fully open access journal, and if your article is accepted for publication, an open access charge will be payable (this does not apply to letters and scientific commentaries). Details can be found in our Instructions for Authors: https://academic.oup.com/braincomms/pages/General_Instructions

Once again, thank you for submitting your manuscript to Brain Communications and I look forward to receiving your revision.

Best wishes

Dr. Alejandro Higuera-Matas
Associate Editor, Brain Communications

Prof. David Belin
Editor-in-Chief Brain Communications

Reviewer(s)' Comments to Author:

Reviewer: 1

Comments to the Author

Please delete the word "simply" from the abstract and discussion. For instance, "...challenging the paradigm of simply reducing Aβ burden" implies that reducing Aβ burden ALONE is not sufficient, as it must be COMBINED with something else. This is inaccurate. A minor but more accurate conclusion is "This work establishes Aβ42 as a physiological neurometabolic coupler in the PFC and has direct clinical implications for AD: it supports therapeutic strategies aimed at preserving or restoring physiological Aβ42 levels rather than further reducing them".

Reviewer: 2

Comments to the Author
The authors have correctly addressed all my comments.

Comments from the Scientific Editorial Office:

- Thank you for including a graphical abstract; however, we have one final minor request. Please clarify whether any external software was used (e.g. BioRender, etc.) to generate the graphics within the abstract. If BioRender was used, for instance, please include an acknowledgement at the bottom of the graphical abstract ("created using BioRender"), accompanied by a link to where the graphics can be found on the BioRender website.

- Please clarify whether any code was generated for the purpose of statistical analysis (e.g. using programs such as R, MATLAB, Python or otherwise). Please note that, if any analysis code was generated, we strongly encourage authors to make such code available to readers, either via online repository such as GitHub or attached directly as supplementary materials to the main manuscript.

- Figures 1 and 3: please include a statement in the figure legend to indicate what each datapoint/dot in the bar chart represents (e.g. "each datapoint represents an independent measure from a single experiment/passage/determination" or otherwise).

- Figures 2A and 3A: please note that, where N < 10 for any group, we request that individual datapoints be shown in plots. Please show datapoints overlaid atop the bar charts in a similar manner to that of Figure 1. When including datapoints, please also state what each point represents in the figure legend. Finally, please spell out remaining abbreviations in the figure legend, such as "PFC".

- Figures 2B and 3B: please note that we require full, uncropped images of gels to also be included as supplementary material. Please include the uncropped version of these gels as supplementary figures.
