# Supplementary material for: Activity-dependent glucose uptake in the mouse prefrontal cortex depends on endogenous beta-amyloid production
Source: Brain Commun. 2026 Oct 28;8(6):fcag412. doi: 10.1093/braincomms/fcag412 (PMC13739058; doi:10.1093/braincomms/fcag412)
Supplement: Supplementary file 1 [file fcag412_supplementary_data.zip › Supplementary_Material.pdf]

Supplementary Material

Activity-dependent glucose uptake in the mouse prefrontal cortex depends on endogenous Aβ production

Short title: Endogenous Aβ couples activity to glucose uptake

Annamaria Fidilio[1], Sofia Varrasi[1,2], Rosa Santangelo[1], Filippo Caraci[1,3], Agata Copani[1,4]

[1]Department of Drug and Health Sciences, University of Catania, 95125 Catania, Italy.
[2]PhD Program in Neuroscience, University of Catania, Italy.
[3]Oasi Research Institute-IRCCS, Unit of Neuropharmacology and Translational Neurosciences, 94018 Troina, Italy.
[4]Institute of Crystallography, National Research Council, 95126 Catania, Italy.

Correspondence to: Agata Copani, Department of Drug and Health Sciences, University of Catania, 95125 Catania, Italy. Tel: + 39(095)7384212. E-mail: acopani@katamail.com.

Full images of Figure 2B WBs

Odyssey Image

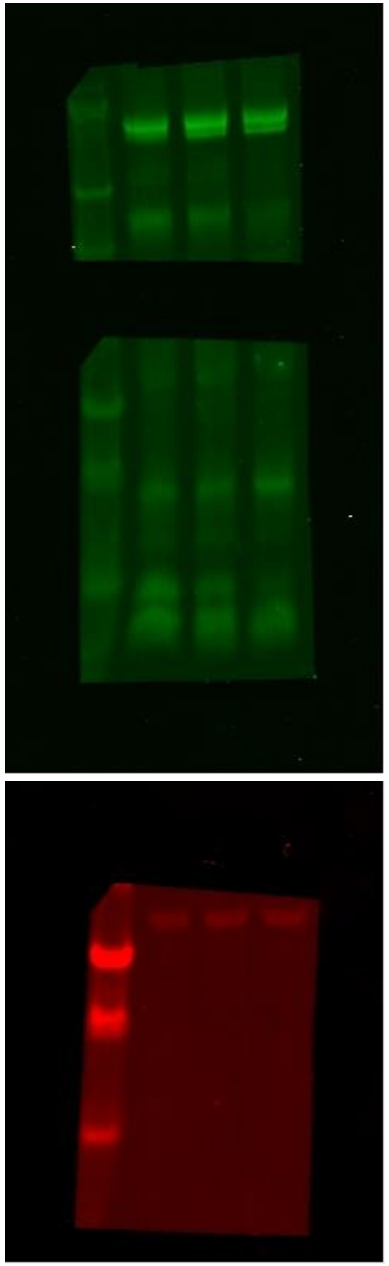

The membrane was cut into two parts for antibody incubation

Grey Level Image

Holo APP

198 kD -

38 KD -

APP-CTFs

14 KD -

β–actin

38 KD -

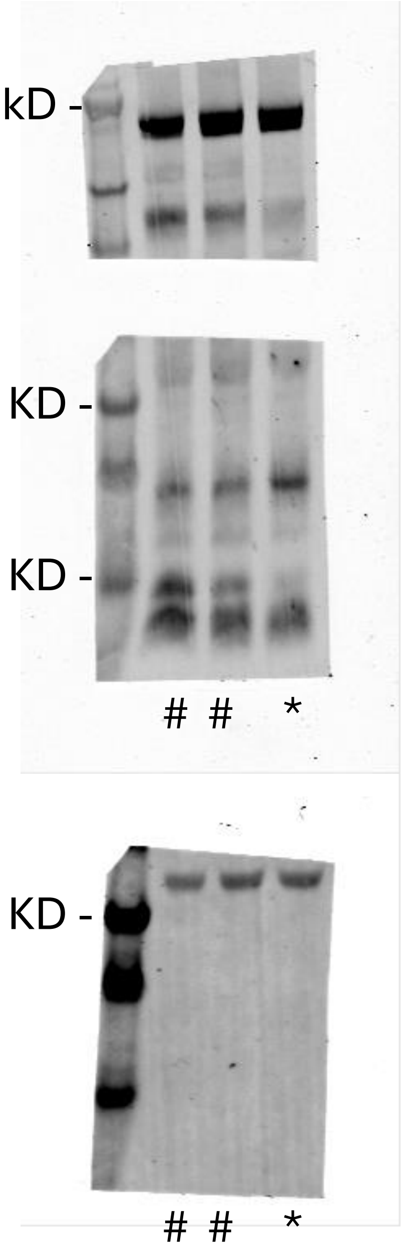

Shown in the manuscript

* Not relevant to this study

Full images of Figure 3B WBs

Odyssey Images

Grey level Images

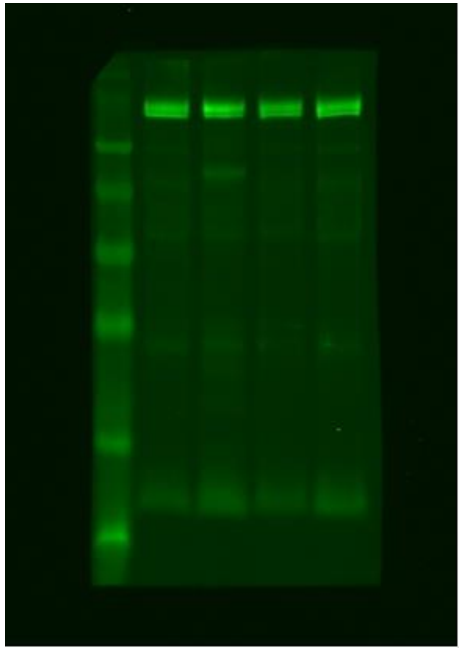

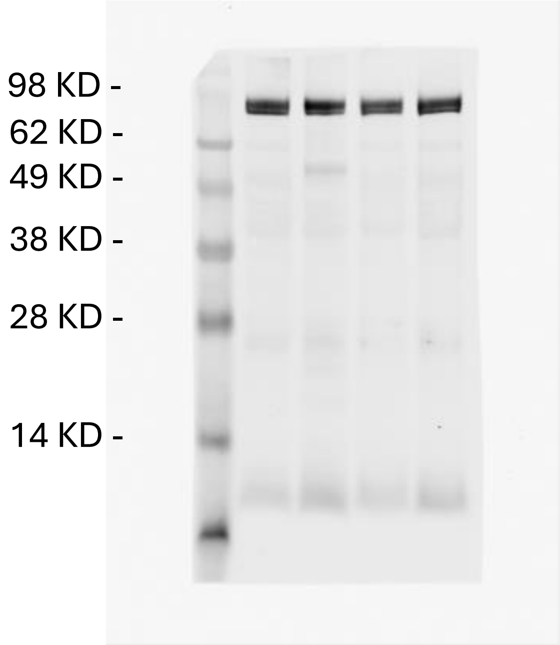

The membrane was cut at 28 kD and re-blotted with primary and secondary antibodies to improve the signal below 28 KD

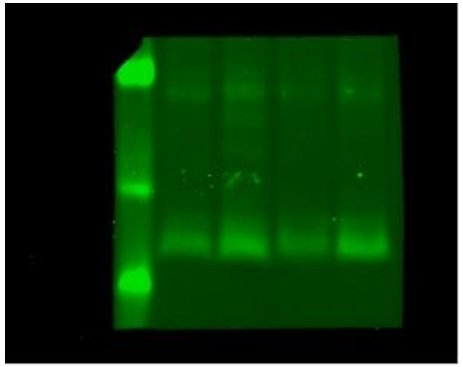

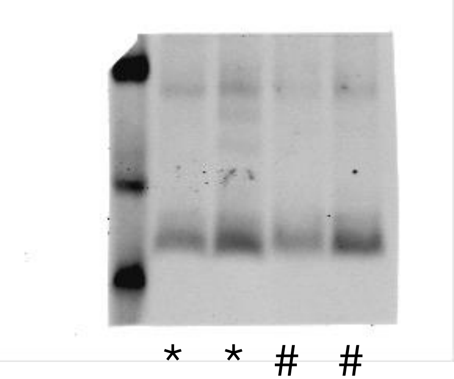

* Not relevant to this study
# Shown in the manuscript
